# Supplementary material for: Stacking faults from a different angle – Overcoming the edge-on limit in high-resolution defect analysis
Source: Nat Commun. 2026 Sep 11;17:9744. doi: 10.1038/s41467-026-77584-z (PMC13569835; doi:10.1038/s41467-026-77584-z)
Supplement: Supplementary file 1 [file 41467_2026_77584_MOESM1_ESM.pdf]

Supplementary Information

Stacking faults from a different angle – Overcoming the edge-on limit in high-resolution defect analysis

Nicolas Karpstein [a], Lukas Müller [a], Andreas Bezold [b,c], Michael J. Mills [c], Steffen Neumeier [b], Erdmann Spiecker [a,*]

[a] Friedrich-Alexander-Universität Erlangen-Nürnberg, Department of Materials Science & Engineering, Institute of Micro- and Nanostructure Research, and Center for Nanoanalysis and Electron Microscopy (CENEM), IZNF, Erlangen, Germany

[b] Friedrich-Alexander-Universität Erlangen-Nürnberg, Department of Materials Science & Engineering, Institute I: General Materials Properties, Erlangen, Germany

[c] The Ohio State University, Department of Materials Science & Engineering, Columbus, OH, USA

* Corresponding author: erdmann.spiecker@fau.de

<u>Supplementary Note 1</u>. Stacking fault types and corresponding lattice translations in the fcc and $L1_2$ crystal structures.

The fcc and $L1_2$ crystal structures give rise to various SF types, which will be outlined in the following (Supplementary Fig. 1). To visualize the lattice translation associated with each type of stacking fault exemplarily in the (111) glide plane, a stack of three (111) planes is shown where the bottom, middle, and top layer are represented by small, medium-sized and large atoms, respectively. Arrows represent the translation of the top layer against the lower ones required to form a given fault.

In the fcc structure (Supplementary Fig. 1a), $a/6\,\langle 211\rangle$ Shockley partial dislocations introduce intrinsic SFs (ISFs) into the crystal structure (green arrows) and make it appear as though a plane is missing from the …ABCABC… stacking sequence of close-packed planes. As evident from the illustration, the corresponding translation can occur in three different directions; however, all three translation vectors result in crystallographically identical fault structures. In other words, the difference vector between any two of these vectors is equal to a lattice translation vector. An extrinsic SF (ESF) consists of two ISFs on neighboring glide planes, resulting in net translations of type $a/3\,\langle 211\rangle$ as illustrated by purple arrows. In this case, the stacking sequence appears to have an extra plane inserted into it. For simplicity, the arrows representing two-layer faults in Supplementary Fig. 1 are drawn in one crystal plane to visualize the total shifts between the upper and lower half crystal.

In the $L1_2$ structure (Supplementary Fig. 1d), the superstructure gives rise to additional distinctions between fault types based on whether nearest-neighbor violations (i.e., Al sites being unfavorably close to each other) exist between the fault planes. Intrinsic and extrinsic SFs still have the same effect on the stacking sequence as in the fcc structure. However, a Shockley partial now introduces a complex intrinsic SF (CISF, light green arrows) with additional nearest-neighbor violations. For a superlattice intrinsic SF (SISF, dark green arrows) without such violations, a twice as large $a/3\,\langle 211\rangle$ Kear partial is required. Again, in both cases, this translation is possible in three different directions, and in the case of the SISF, all three translation directions lead to identical fault structures. On the other hand, the three CISF are crystallographically distinct, albeit only in their superstructure. Analogous to the fcc structure, a complex extrinsic SF (CESF, light purple arrows, also referred to as CESF-2) can be described as comprising two identical CISFs on neighboring planes. Similarly, a superlattice extrinsic SF (SESF, dark purple arrows) consists of two neighboring SISFs. Notably, the net translation

vectors of CESF and SESF are identical; the two faults differ only in the translation of their central plane. Consequently, a CESF can be transformed into an SESF by local reordering of the central fault plane, as postulated by the Kolbe mechanism (see main text for details).

Supplementary Note 2. Projections of fault translation vectors along the [110] and [001] viewing directions.

For the projections of the net translation vectors of faults along the [110] and [001] viewing directions, the faults are considered to be situated on the (111) plane in accordance with Supplementary Fig. 1a,d and the main text, without loss of generality. Likewise, the structures in Supplementary Fig. 1b,c,e,f are rotated so that the depth of the SF and its (111) habit plane increases from left to right. In [110] projection, this means that the [001] direction points to the right, whereas in [001] projection, the [110] direction points to the right.

Projected net translation vectors of ISF and ESF in the fcc structure are shown in Supplementary Fig. 1b,c.

In the [110] ZA (Supplementary Fig. 1b), the projections of the ISF translation vectors $^a/_6\,[11\bar{2}]$, $^a/_6\,[1\bar{2}1]$, and $^a/_6\,[\bar{2}11]$ are $^a/_3\,[\mathit{00\bar{1}}]$, $^a/_{12}\,[\mathit{3\bar{3}2}]$, and $^a/_{12}\,[\mathit{\bar{3}32}]$, respectively (for better distinction, projected vectors are written in italics). Since all three ISF translation vectors result in the same fault structure, the same holds true for the projected vectors (summarized as $^a/_3\,[\mathit{00\bar{1}}]$ in the main text) and resulting structure, where the shifted lattice appears translated by one third of the periodicity to the left of the reference lattice (Fig. 2a, left).

The ESF translation vectors and thus their respective projections are twice as large. Again, the resulting three projected fault structures are identical (and summarized as $^{2a}/_3\,[\mathit{00\bar{1}}]$ in the main text). Here, the shifted lattice appears to the right of the reference lattice (Fig. 2a, left).

In the [001] ZA (Supplementary Fig. 1c), the ISF translation vectors $^a/_6\,[11\bar{2}]$, $^a/_6\,[1\bar{2}1]$, and $^a/_6\,[\bar{2}11]$ have projections of $^a/_6\,[\mathit{110}]$, $^a/_6\,[\mathit{1\bar{2}0}]$, and $^a/_6\,[\mathit{\bar{2}10}]$, respectively, with identical projected fault structures (the projected translation is summarized as $^a/_6\,[\mathit{110}]$ in the main text). This means that the shifted lattice appears to the right of the reference lattice (Fig. 2a, right).

Again, the three translation vectors of ESFs and their projections are twice as large and the three projected structures identical (summarized as $^{a}/_{3}\,[110]$ in the main text) with the shifted lattice appearing to the left of the reference lattice (Fig. 2a, right).

Projections of SISF and SESF translation vectors in the $L1_2$ structure are illustrated in the same way in Supplementary Fig. 1e,f. Compared to the ISF in the fcc structure, translation vectors of the SISF – and thus their projections in the [110] and [001] ZAs – are twice as large and antiparallel, also leading to projected fault structures where the shifted lattice appears to the left of the reference lattice in the [110] ZA (Fig. 2b, left) and to the right in the [001] ZA (Fig. 2b, right). The net translation vectors of SESFs in $L1_2$ are identical to those of ESFs in fcc, and the same applies to their projections. In the projected fault structures, the shifted lattice appears to the right of the reference lattice in the [110] ZA (Fig. 2b, left) and to the left in the [001] ZA (Fig. 2b, right).

<u>Supplementary Note 3</u>. Projected structures of complex faults in the $L1_2$ structure.

The difference between complex faults and their non-complex (superlattice) counterparts in the $L1_2$ structure is only in the superlattice ordering, i.e., the stacking sequence of close-packed planes, ignoring the individual sublattices, is the same. Therefore, atomic columns also appear in the same positions in HRSTEM micrographs; differences in projected fault structures can only arise in terms of which atomic columns appear brighter or darker due to Z contrast.

The projected fault translation vectors of CISFs, along with SISFs for comparison, are presented in Supplementary Fig. 2 (cf. Supplementary Fig. 1b for the in-plane view of fault translation vectors) along with HAADF-STEM multislice simulations of the corresponding faults. Unlike their superlattice counterparts, the three possible translation directions result in crystallographically distinct fault structures, again differing only in their superstructure. Thus, all three are considered separately and numbered for reference.

In the [110] ZA (Supplementary Fig. 2a), the projected fault translation vector of $^{a}/_{3}\,[00\bar{1}]$ results in a projected fault structure identical to that of an SISF (cf. Fig. 6 in the main text), where neighboring columns are of the same type and thus appear at the same brightness in HAADF contrast (Supplementary Fig. 2c). The projected translation vectors $^{a}/_{12}\,[3\bar{3}2]$ and

$^{a}/_{12}\,[\bar{3}32]$, on the other hand, result in a different projected structure where mixed neighboring columns appear. Thus, only in these two cases, the CISF can unambiguously be identified as such. As a visual guide, brighter columns are highlighted by yellow circles.

A similar pattern emerges in the [001] ZA (Supplementary Fig. 2b,d): the projected structure associated with $^{a}/_{6}\,[110]$ is identical to that of the SISF, recognizable by the presence of $(1\bar{1}0)$ planes (i.e., horizontal rows) containing only one sublattice. The other two projected translation vectors, $^{a}/_{6}\,[1\bar{2}0]$ and $^{a}/_{6}\,[\bar{2}10]$, result in projected structures characteristic to the CISF.

CESFs are not distinguishable at all from SESFs using the method presented in this work. This is because the contrast of projected fault structures almost only depends on the net translation vector of the imaged inclined fault, which is identical for CESF and SESF (cf. Supplementary Fig. 1d). Between the two fault structures, the superstructure only of the central fault plane is shifted. This difference within a single crystal plane has a negligible effect on image contrast.

Supplementary Figures

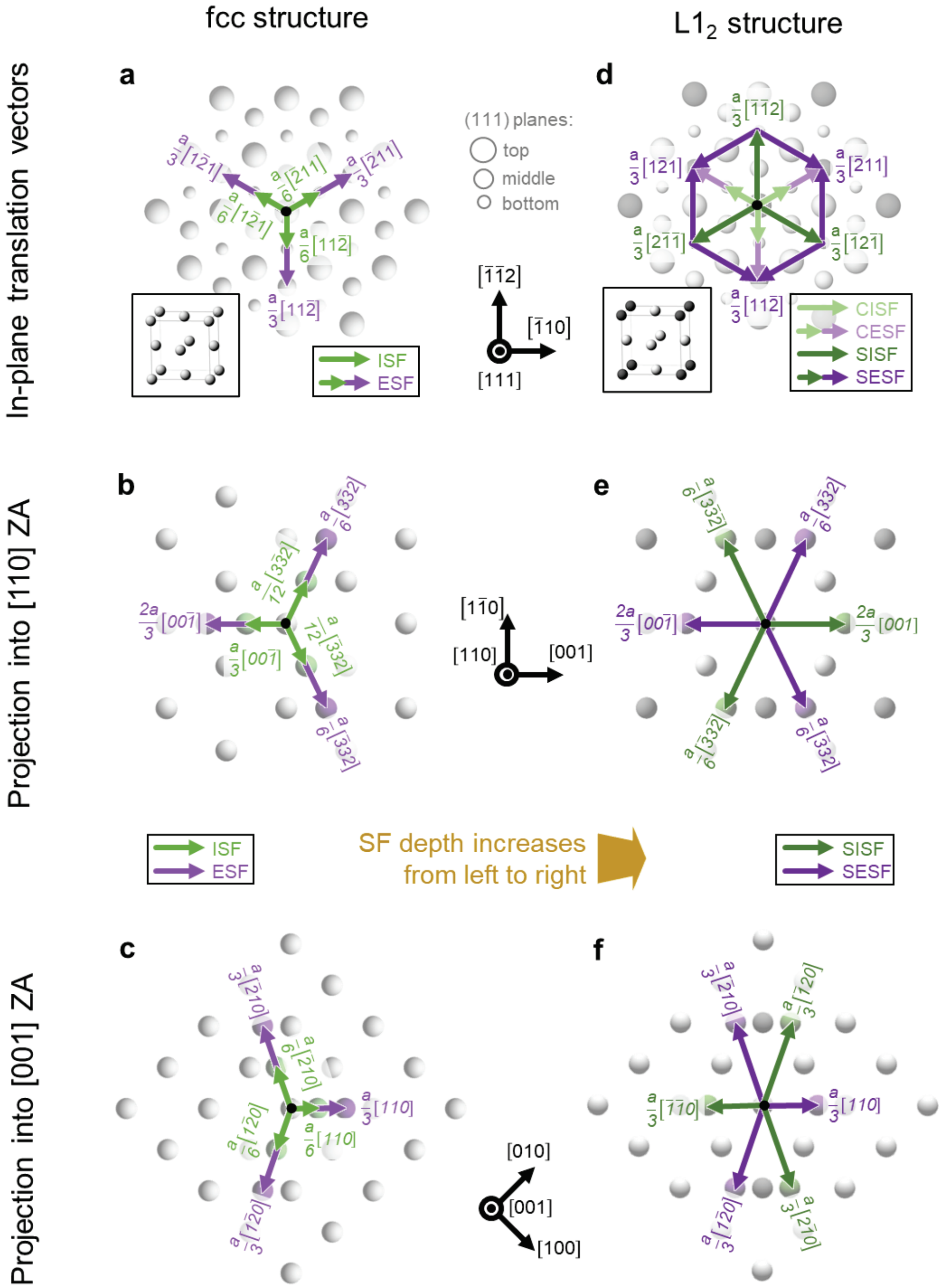

Supplementary Fig. 1: SF types, their translation vectors and their [110] and [001] projections, in the (a-c) fcc and (d-f) $L1_2$ structure. (a,d) Schematic illustrations of a stack of three (111) planes in the (a) fcc and (d) $L1_2$ structure. Unit cells are shown as an inset. The vectors by which the top half-crystal is translated to form a given fault are represented by arrows colored depending on the fault type. Schematic illustrations of the fcc and $L1_2$ crystal structures in (b,e) [110] and (c,f) [001] projection, with net translation vectors of (S)ISF and (S)ESF projected into the respective plane. The projected vectors correspond to those shown in (a,d), i.e., to a fault on the (111) plane. In accordance with the analysis presented in the main text, the structures are rotated so that the depth of the (111) plane inclined to the viewing direction increases from left to right.

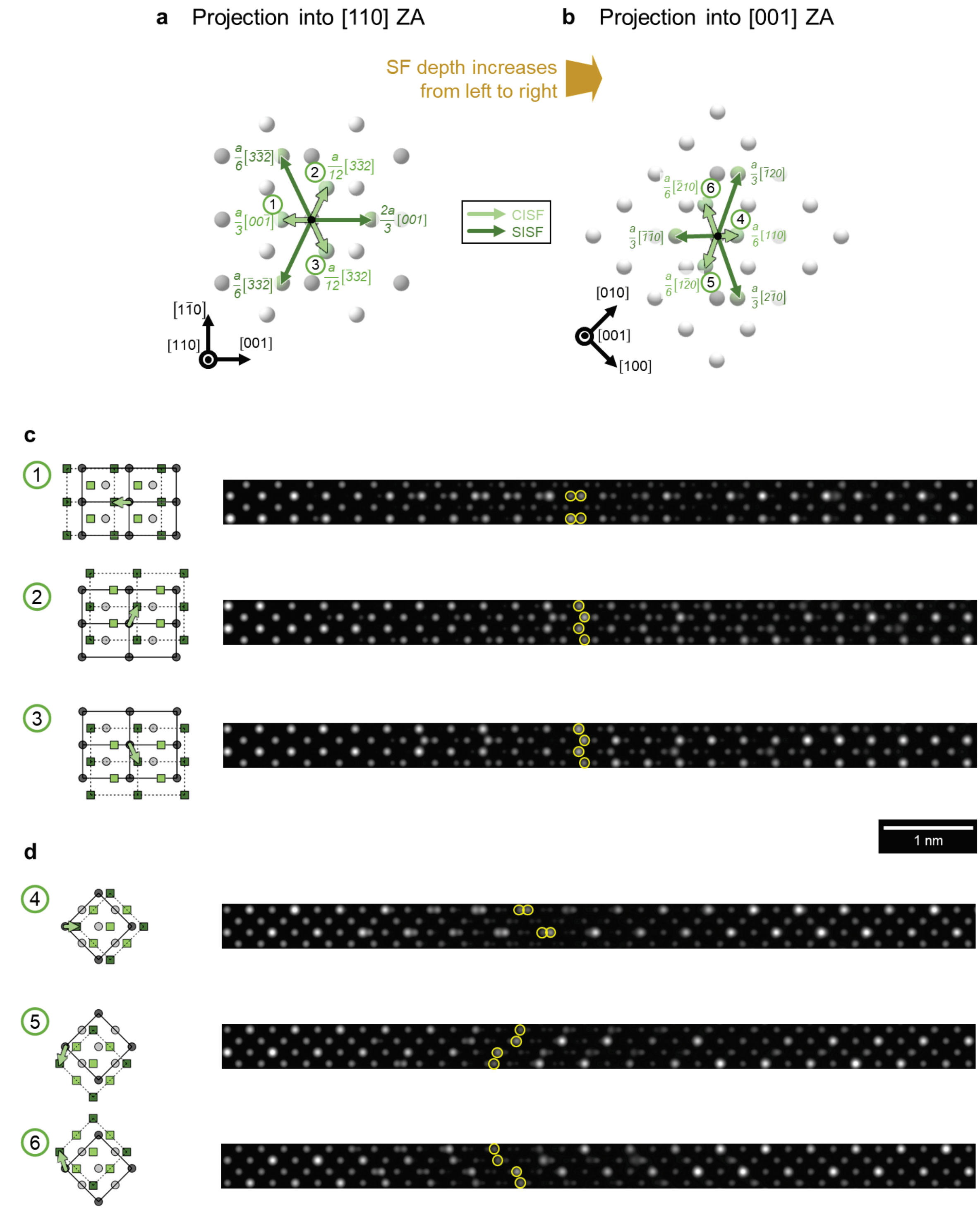

Supplementary Fig. 2: Schematic illustration of the $L1_2$ crystal structure in (a) [110] and (b) [001] projection, with net translation vectors of CISF (and SISF for comparison) projected into the respective plane. The projected vectors correspond to those shown in Supplementary Fig. 1d, i.e., to a fault on the (111) plane. In accordance with the analysis presented in the main text, the structures are rotated so that the depth of the (111) plane inclined to the viewing direction increases from left to right. Below, projected structures and multislice image simulations (for a reduced sample thickness of 12 nm for enhanced superlattice contrast) corresponding to the different CISF translation vectors are shown for (c) [110] and (d) [001] projection.

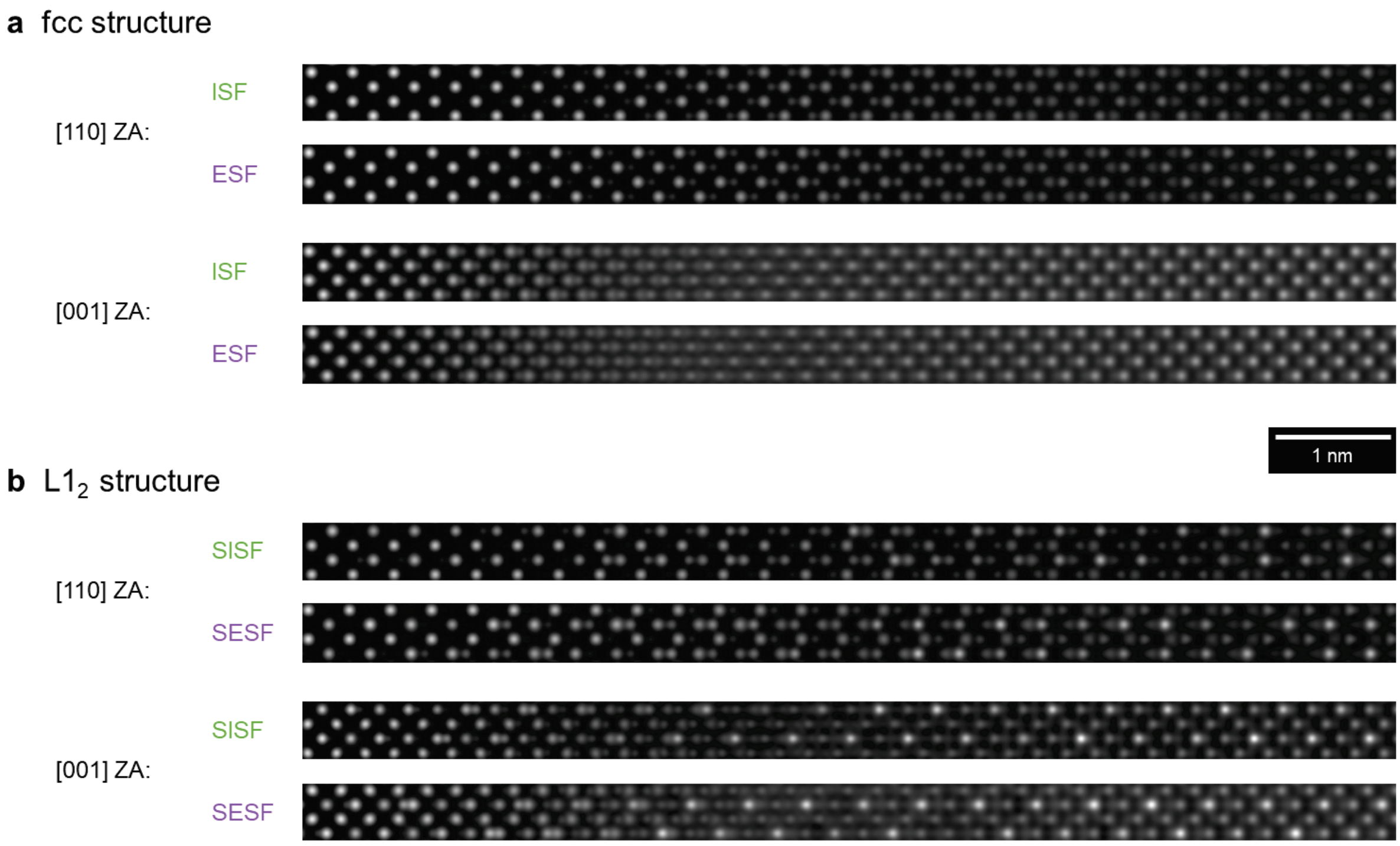

Supplementary Fig. 3: HAADF-STEM multislice simulations of inclined SFs for the different fault types in (a) fcc structure and (b) $L1_2$ structure. Foil thickness ≈30 nm in all cases.

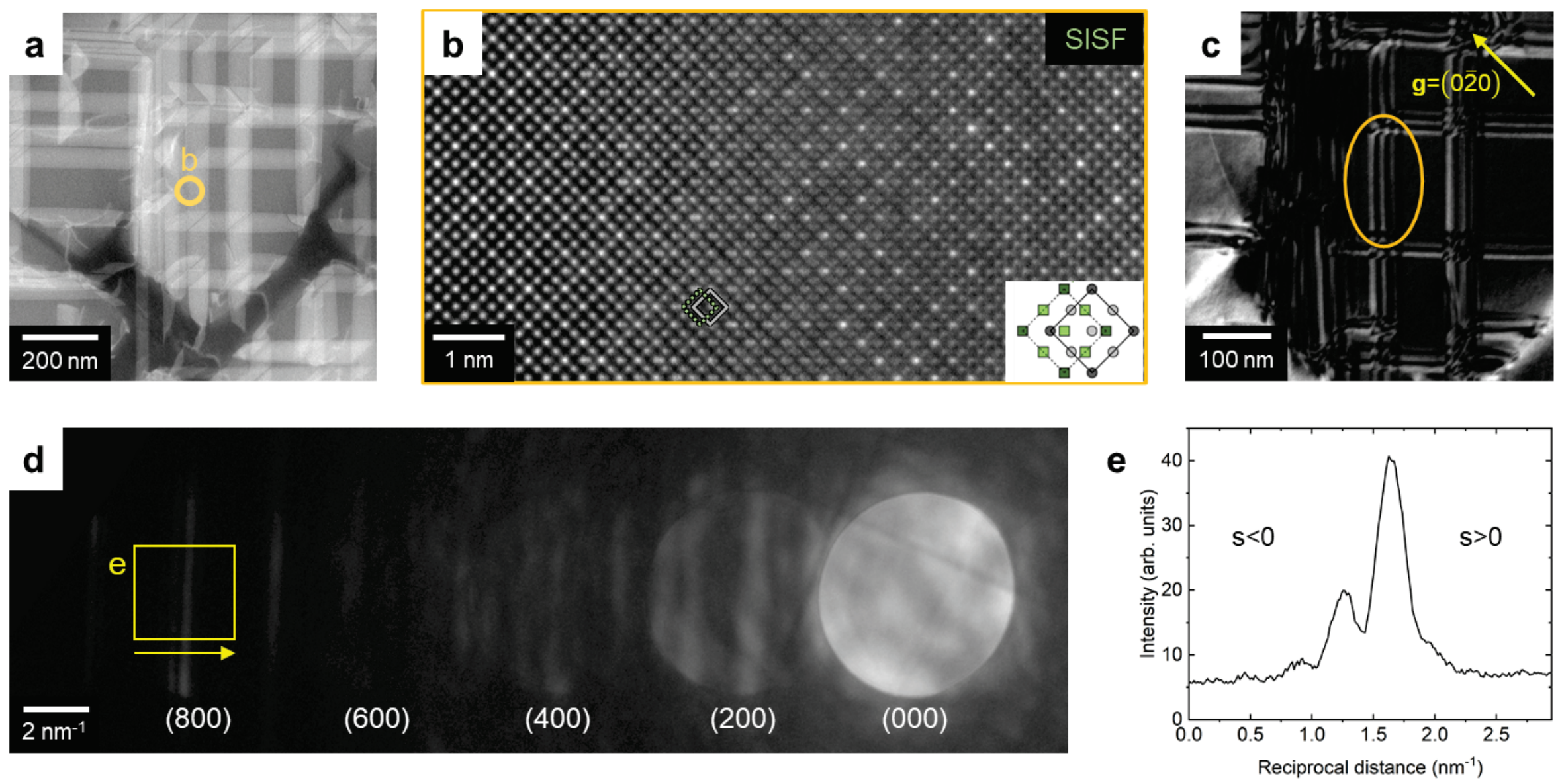

Supplementary Fig. 4: Analysis of the same SF using three different methods. (a) ADF-STEM micrograph of the surrounding microstructure. (b) HRSTEM micrograph of the top edge of the fault marked in (a), revealing it to be intrinsic. (c) Dark-field TEM micrograph of the same fault in a $(0\bar{2}0)$ two-beam condition. **g** points towards the bright outer fringe, confirming the fault's intrinsic character. (d) Energy-filtered CBED pattern with the beam converged on the center of the same fault and the (800) reflection excited. (e) Intensity profile of the region marked in (d), clearly revealing splitting of the (800) Bragg line. The additional line of lower intensity appears in the direction of negative excitation error, indicating a phase shift of $-{}^{2\pi}/_{3}$ and thus an intrinsic fault.

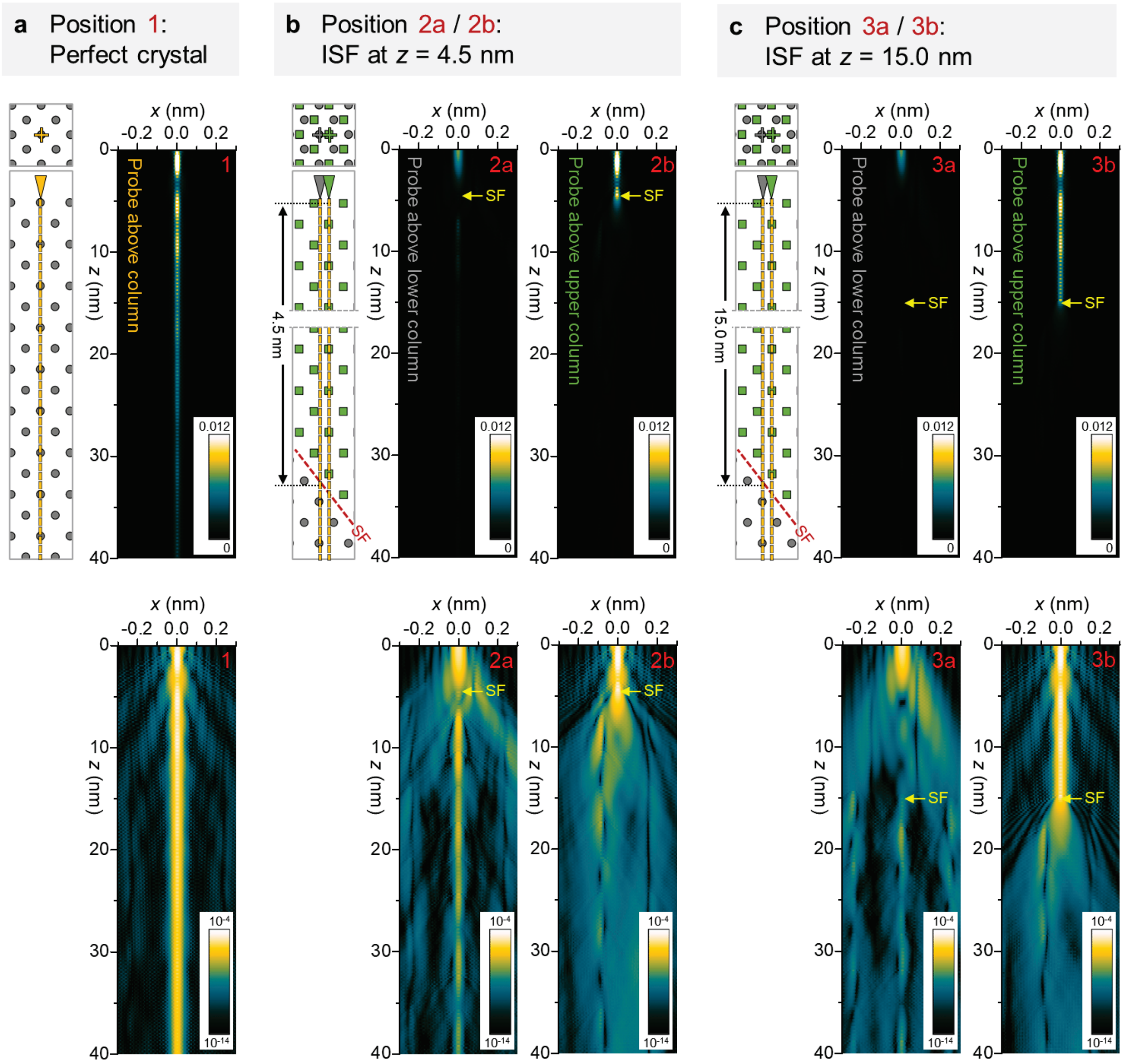

Supplementary Fig. 5: Linear (top) and logarithmic (bottom) presentation of the slices of simulated probe intensity distributions shown in Fig. 4 in the main text.

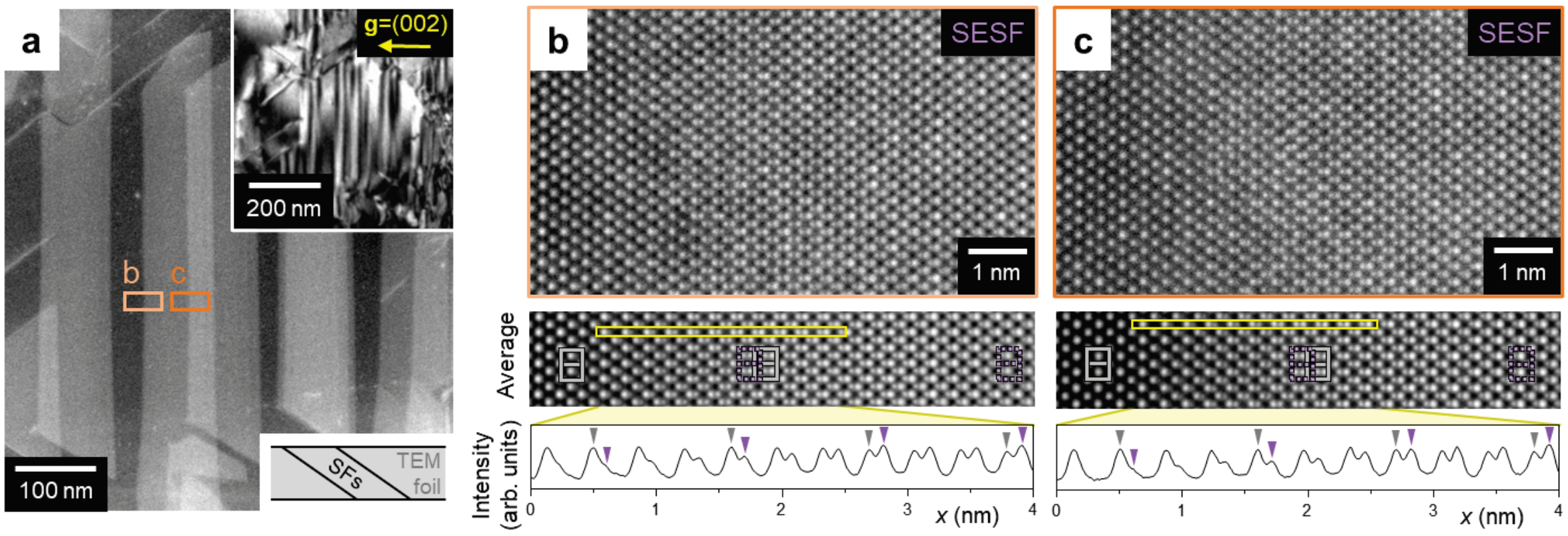

Supplementary Fig. 6: Analysis of SFs overlapping in projection. (a) ADF-STEM micrograph of overlapping SFs in the [110] ZA. The inset at the top right shows a dark-field TEM image of the same region with fringe contrast difficult to interpret. (b,c) HRSTEM micrographs of the locations marked by rectangles in (a), from which both faults can be analyzed separately and identified as extrinsic. Below each HRSTEM micrograph, the periodically averaged signal is shown along with intensity linescans from the areas marked by yellow rectangles. In the intensity profiles, some peaks of the reference and shifted lattice are highlighted by grey and colored triangles, respectively.

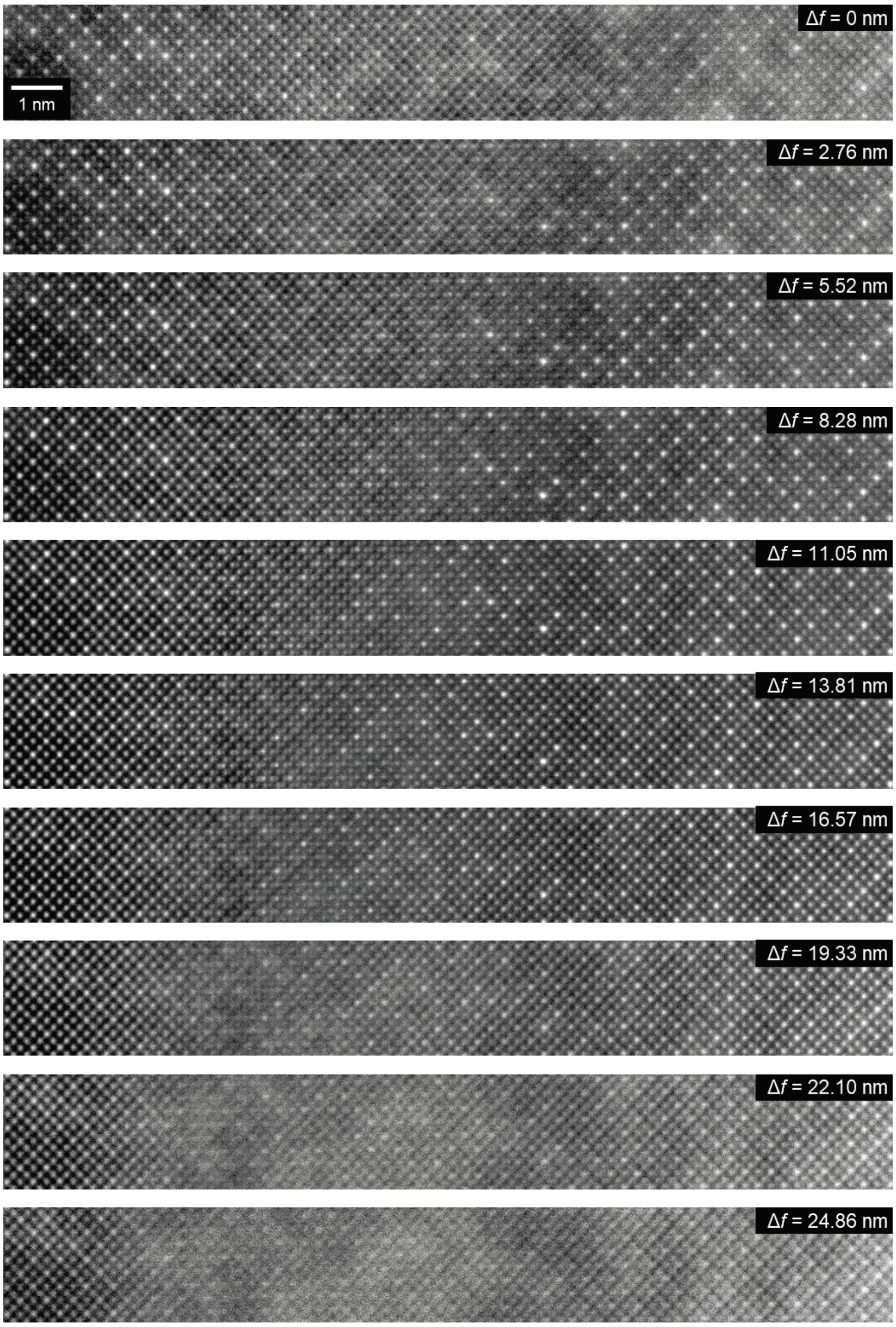

Supplementary Fig. 7: HRSTEM micrographs of the same specimen region containing the top edge of an inclined SISF, under varying defocus Δf (arbitrary reference value) and at a constant beam semi-convergence angle α of 30 mrad. With increasing overfocus (higher Δf), the thickness range sampled by atomic-column contrast is shifted more towards the top of the foil, making the region of overlap between reference and shifted lattice appear further to the left (and vice versa for increasing underfocus).

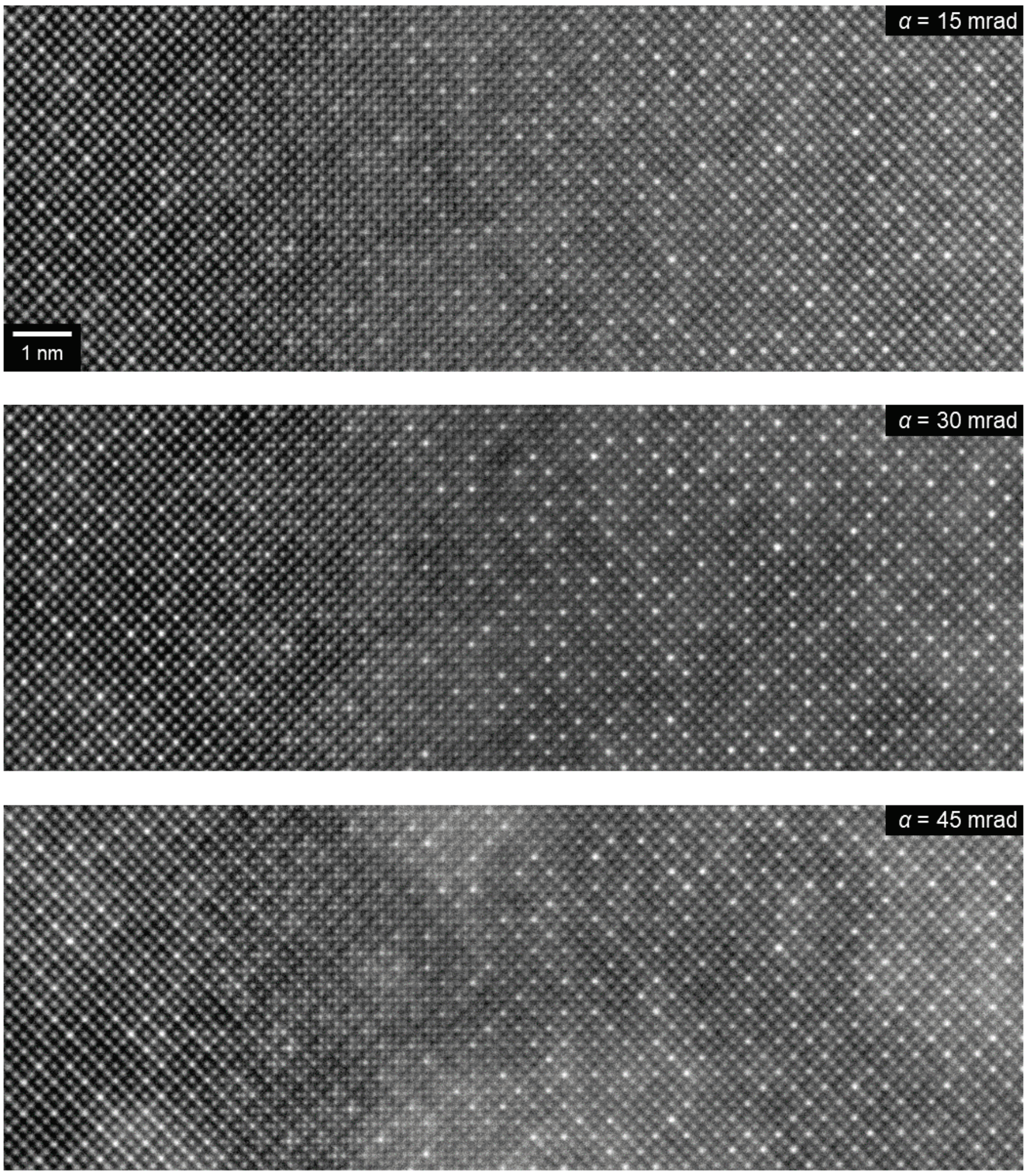

Supplementary Fig. 8: HRSTEM micrographs of the same specimen region containing the top edge of an inclined SISF, under varying beam semi-convergence angle α (achieved by changing the C2 aperture). The higher α, the lower the depth of field; thus, the region of visible overlap between reference and shifted lattice becomes narrower. Note that no re-tuning of the probe corrector was performed after changing α, leading to an increased effect of aberrations in the lower micrograph.

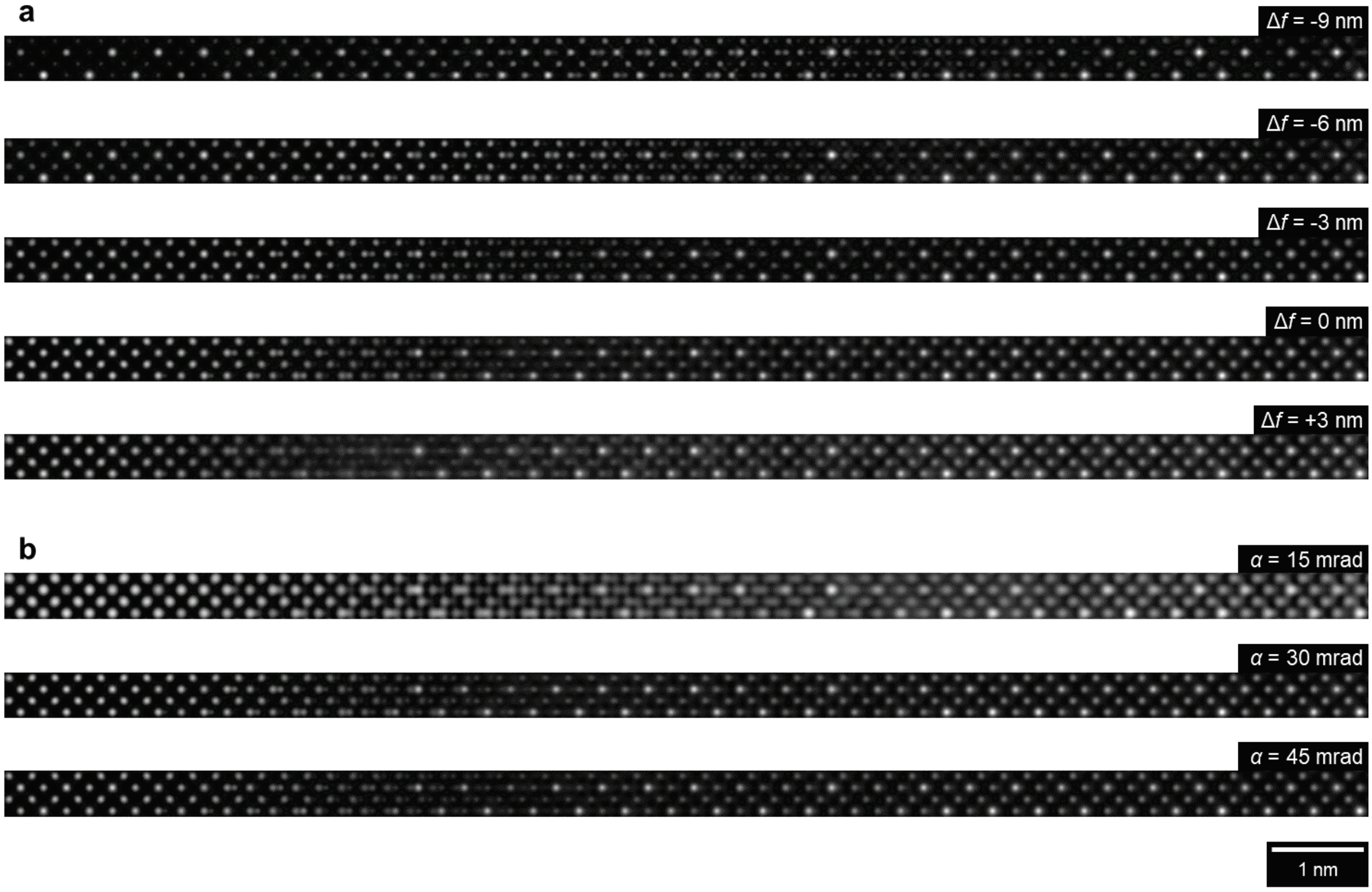

Supplementary Fig. 9: HAADF-STEM multislice simulations of an inclined SISF in $Co_3(Al,W)$ (a) under varying defocus Δf at a constant beam semi-convergence angle of 30 mrad, and (b) under varying beam semi-convergence angle α at a constant defocus of 0 nm. Foil thickness ≈30 nm in all cases.

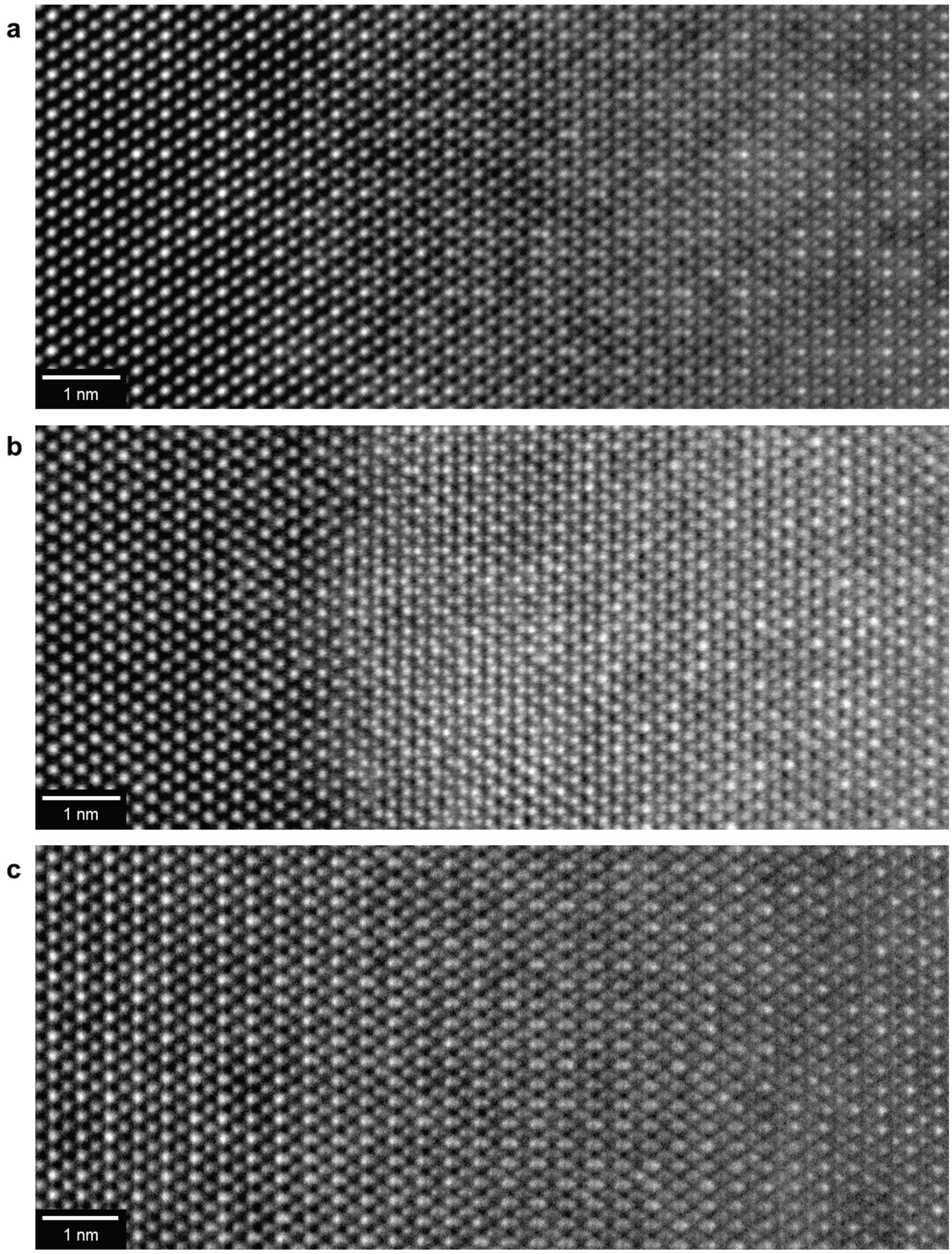

Supplementary Fig. 10: Influence of specimen preparation procedure on HRSTEM contrast of inclined SESFs. HRSTEM micrographs of inclined SFs in foils fabricated using different procedures: (a) electropolishing, (b) electropolishing with subsequent argon ion polishing; (c) focused ion-beam liftout. In all cases, individual atomic columns can be clearly resolved even in the visible overlap region between reference and shifted lattice. The foil thickness is between 60 and 70 nm in all cases.

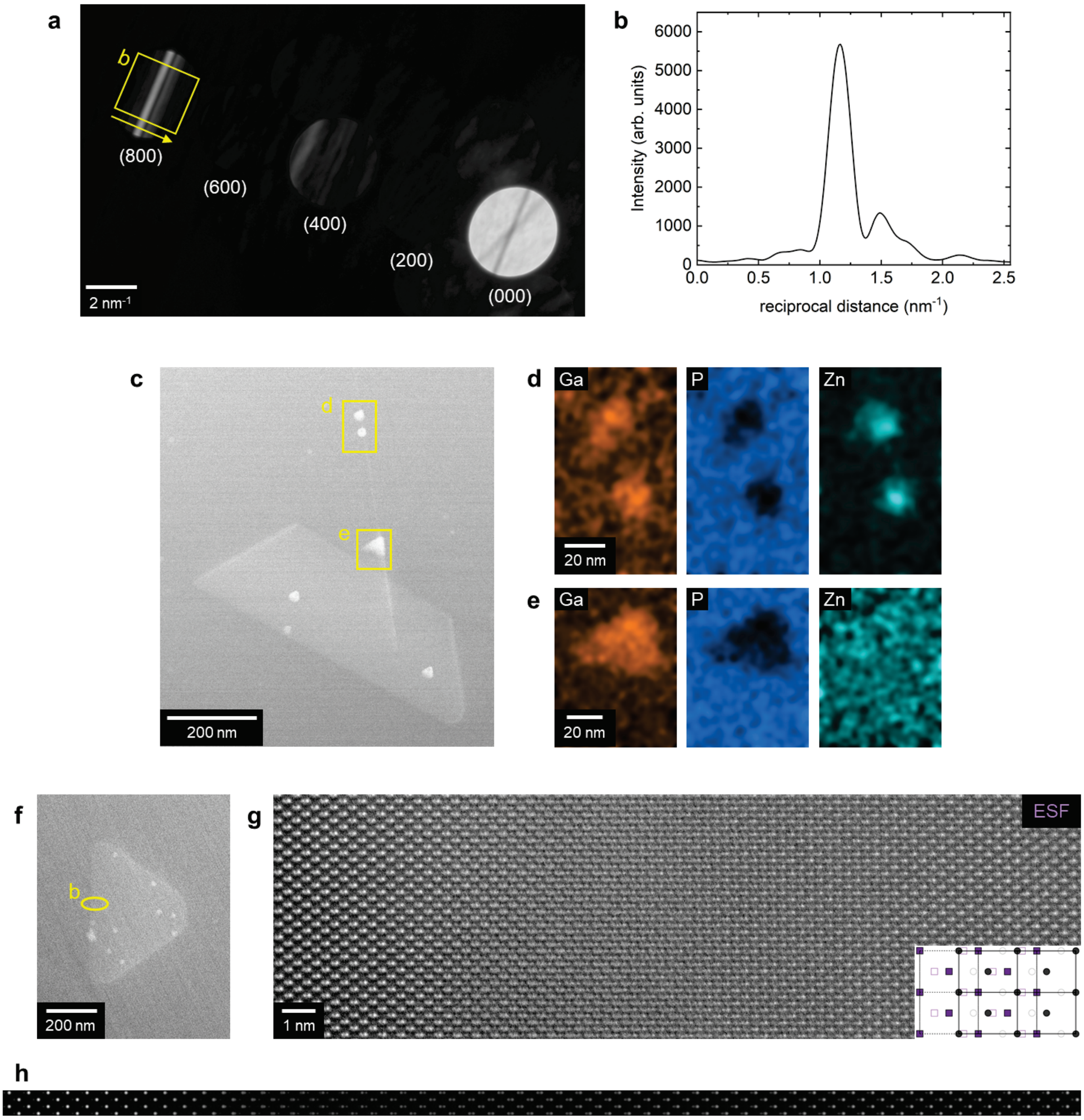

Supplementary Fig. 11: Additional results from the GaP specimen. (a) Energy-filtered CBED pattern with the beam converged on the SF shown in Fig. 8c in the main text, and the (800) reflection excited. (b) Intensity profile of the region marked in (a), clearly revealing splitting of the (800) line. The additional line of lower intensity appears in the direction of positive excitation error, indicating a phase shift of $+{}^{2\pi}/_{3}$ and thus an extrinsic fault. (c) ADF-STEM micrograph of the investigated specimen region. (d,e) EDXS elemental distribution maps (at.%) of the regions marked in (c), showing the elemental distribution in tetragonal voids partially filled with Ga and Zn. (f) Specimen region at a higher thickness of approx. 270 nm with an inclined SF. (g) HRSTEM micrograph of the region marked in (f), revealing the SF to be extrinsic. (h) HAADF-STEM multislice simulation of an inclined ESF in GaP at a thickness of ≈38 nm.

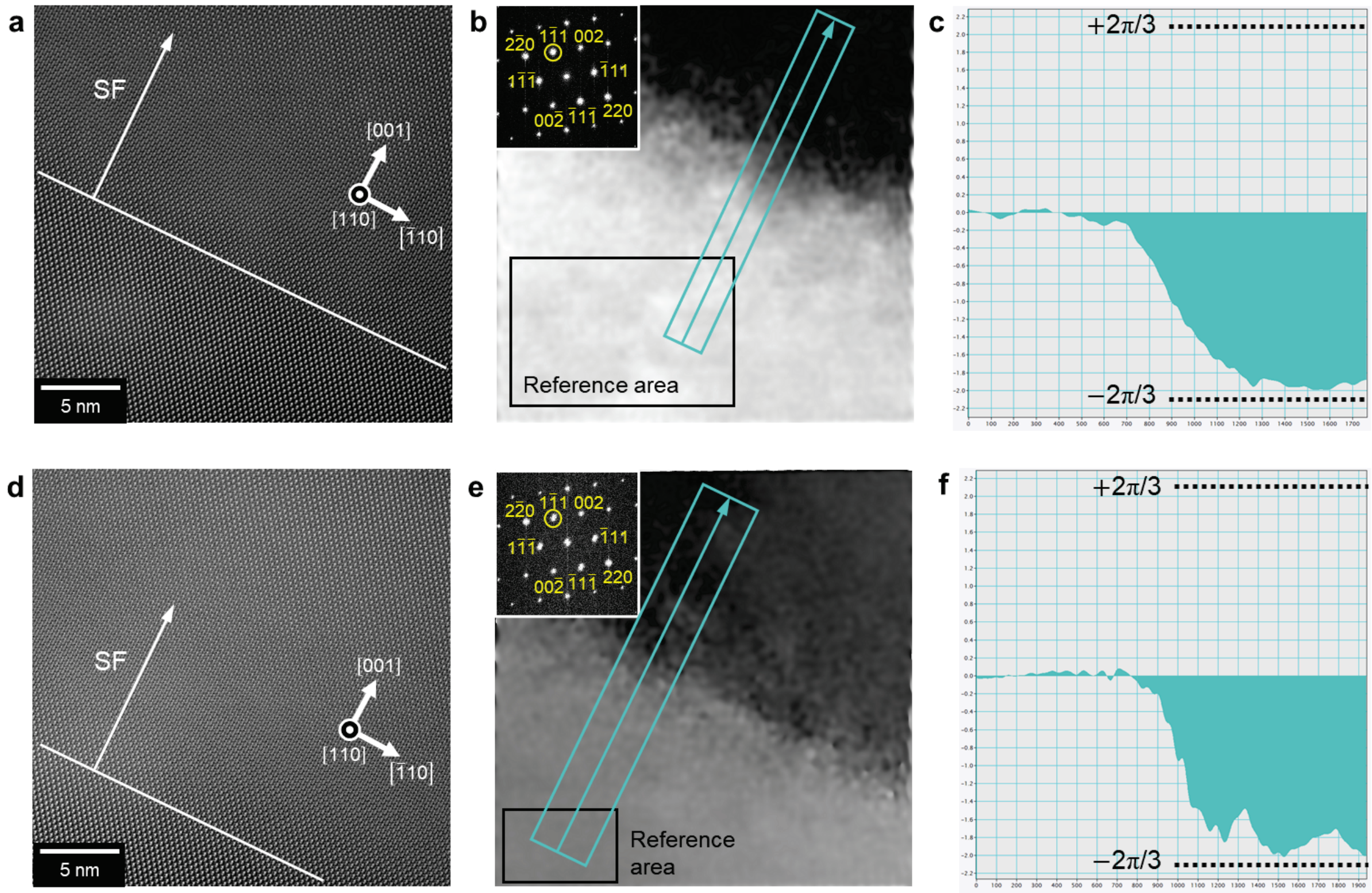

Supplementary Fig. 12: Application of GPA to determine SF type, exemplified by two extrinsic SFs in GaP. (a) HRSTEM micrograph of the inclined SF shown in Fig. 8c in the main text. The top edge of the SF is indicated by the white line. (b) Phase image created with the $(1\bar{1}1)$ reflection as indicated in the Fourier transform of (a) (see inset). The reference area, where the phase shift is defined to be zero, is chosen in the region of perfect lattice before the SF. Across the overlap region, a phase change is visible. (c) Line scan across the overlap region as highlighted in (b), indicating a phase shift towards $-{}^{2\pi}/_{3}$ and thus an extrinsic SF. (d) HRSTEM micrograph of the inclined SF shown in Supplementary Fig. 11g at a foil thickness of approx. 270 nm. (e,f) GPA analogous to the fault above; this SF is also revealed to be extrinsic.

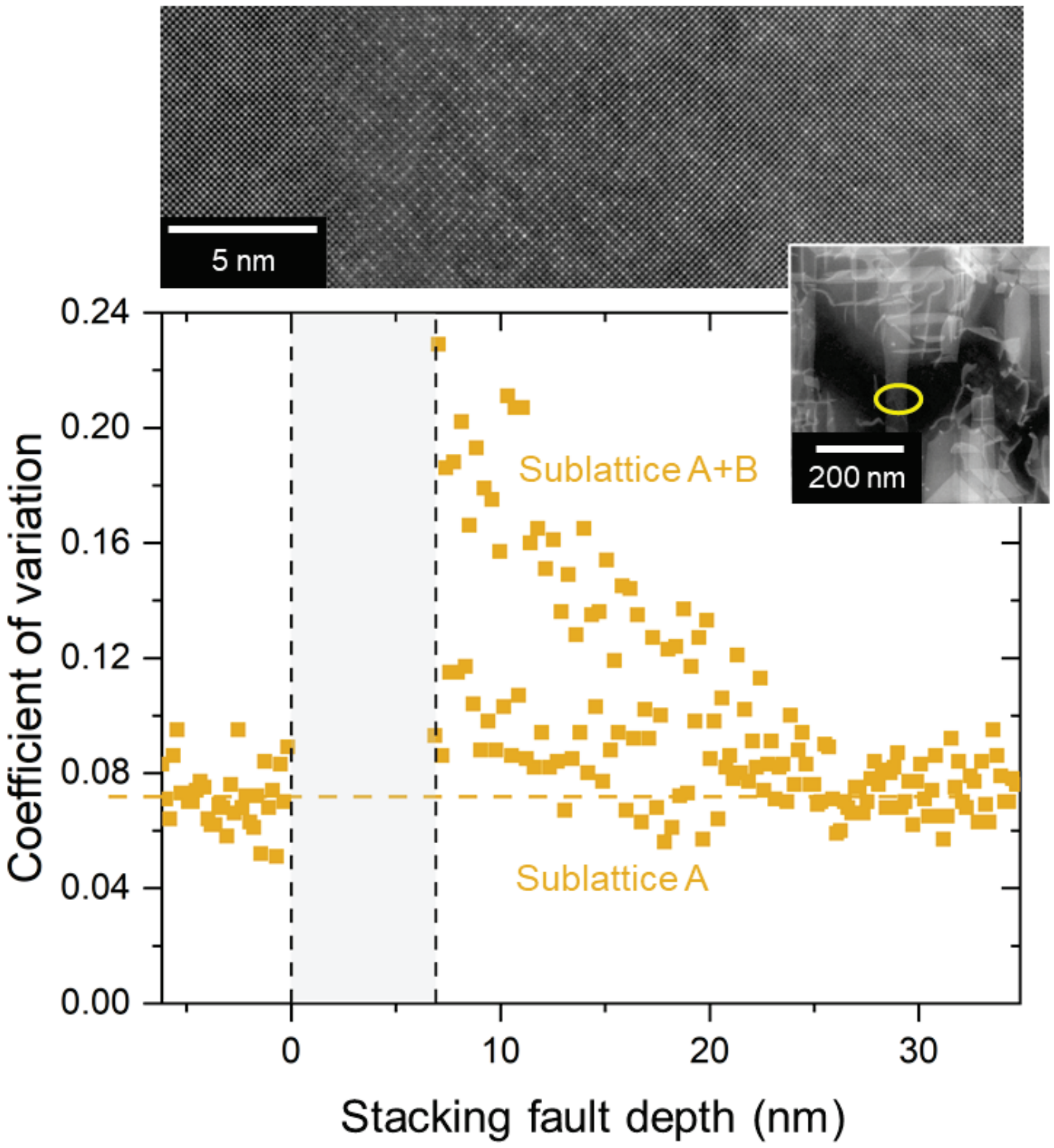

Supplementary Fig. 13: Analysis of enhanced Z-contrast above an inclined SF with a nano-γ′ precipitate. HRSTEM micrograph of an inclined SF in the fcc structure. The plot below shows the CV of atomic-column intensities within each (220) plane. The horizontal line represents the baseline SD determined in a region of perfect lattice to the left of the SF. The HRSTEM image was stitched together from two separate micrographs to obtain a field of view containing regions to the left and right of the inclined SF, from the region shown in the ADF-STEM micrograph (see inset). The nano-γ′ precipitate has a visible width of ≈15 nm.

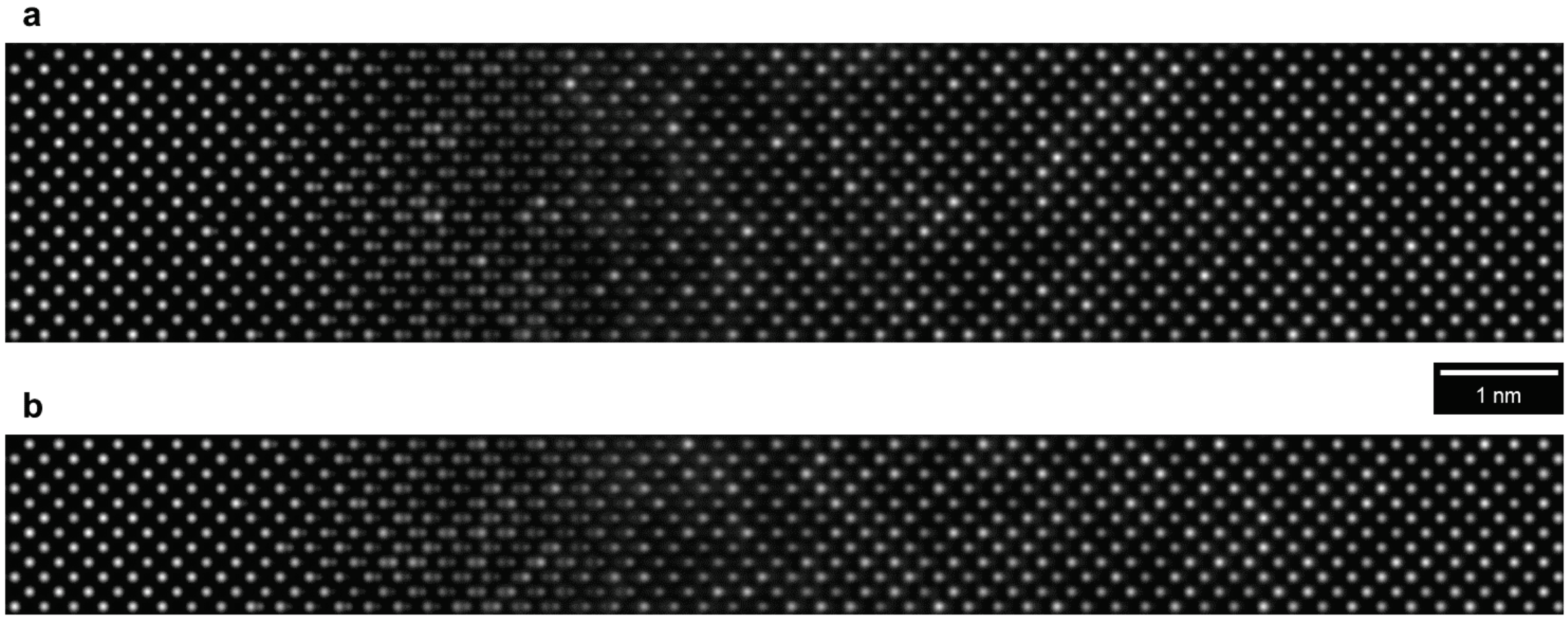

Supplementary Fig. 14: HAADF-STEM multislice simulations of inclined SFs in fcc solid solutions corresponding to the approximate compositions of the γ phase in (a) CoNi-base superalloy ERBOCo-4 ($Co_{50}Ni_{28}W_6Cr_9Ta_2Al_4Ti_1$ in at.%) and (b) Ni-base superalloy CMSX-4 ($Ni_{49}Co_{21}Cr_{18}W_4Re_3Al_4Mo_1$ in at.%). Foil thickness ≈20 nm in both cases.
