# Supplementary material for: Stacking faults from a different angle – Overcoming the edge-on limit in high-resolution defect analysis
Source: Nat Commun. 2026 Sep 11;17:9744. doi: 10.1038/s41467-026-77584-z (PMC13569835; doi:10.1038/s41467-026-77584-z)
Supplement: Supplementary file 2 [file 41467_2026_77584_MOESM2_ESM.pdf]

nature portfolio

Peer Review File

Stacking faults from a different angle - Overcoming the edge-on limit in high-resolution defect analysis

Corresponding Author: Professor Erdmann Spiecker

This manuscript has been previously reviewed at another journal. This document only contains information relating to versions considered at Nature Communications.

This file contains all reviewer reports in order by version, followed by all author rebuttals in order by version.

Version 0:

Reviewer comments:

Reviewer #1

(Remarks to the Author)
This revised version addresses previous comments with additional application examples and discussions, including on the effect of probe focus. Overall, the ability to observe stacking faults both inclined and edge on, as well as being able to indentify the nature of stacking faults in different type of crystal structures is valuable for materials characterization and represents a major use of aberration corrected transmission electron microscopy.

The manuscript is detailed, clearly written. However, the abstract and introduction part can be improved. The robustness of the method is not limited to the CoNi-based superalloy, for example, as the abstract would suggest. I would recommend publication after the authors address this point.

(Remarks on code availability)

Reviewer #2

(Remarks to the Author)
The manuscript presents a HRSTEM-based methodology for analyzing inclined stacking faults in fcc, $L1_2$, and sphalerite crystal structures. By exploiting de-channeling contrast near the top surface of a TEM lamella, the authors demonstrate that the relative lattice translation across an inclined SF can be visualized, enabling discrimination between intrinsic and extrinsic faults. The method is applied to multiple material systems (CoNi-base superalloy, ODS alloy GRX-810, semiconductor GaP) and supported by multislice simulations. The extension to bounding partial dislocations via Burgers circuits and the observation of a "quasi-ultrathin" foil effect are also presented. This study addresses a relevant problem in defect analysis and contains interesting observations. However, in its current form, the manuscript does not yet provide sufficient evidence or discussion to warrant publication in Nature Communications.

Major comments:
1. While the proposed method is promising, several fundamental issues remain unaddressed: the non-unique determination of Burgers vectors, limited applicability, and overstated claims. Unless these concerns are resolved through a clear delineation of the method's capabilities and limitations, the work's impact will remain ambiguous.
2. The authors demonstrate that for partial dislocations bounding a stacking fault, Burgers circuits can be drawn on HRSTEM images to obtain the projected Burgers vector (Fig. 9). However, even within this context, a limitation is not addressed: The Burgers vector obtained from a single zone axis is a two-dimensional projection and is non-unique in three-dimensional space. Different three-dimensional Burgers vectors can yield identical projections along a given zone axis. This ambiguity is inherent to any single-projection analysis, yet the manuscript does not discuss how to resolve it. for example, by acquiring images along multiple zone axes or combining with complementary techniques. Given that the title emphasizes 'overcoming the edge-on limit' in defect analysis, this omission should be addressed.

Minor comments:
3. The method is demonstrated for cubic crystals and their derivatives. However, for HCP, stacking faults are predominantly confined to the basal plane. The authors mention in the rebuttal that the method is principle applicable, but this is not discussed. Does the method inherently rely on the presence of multiple inclined glide planes, and thus on high crystal symmetry? If so, this should be explicitly stated to avoid over-generalization.
4. The authors acknowledge that the method yields only the net translation vector and cannot resolve complex stacking sequences. This is presented as a limitation. However, the manuscript would benefit from a clearer statement that this positions the method as a complementary tool to edge-on HRSTEM, not a replacement. For studies requiring atomic-scale stacking sequences or chemical ordering within the fault plane, edge-on analysis remains indispensable. Framing this as a complementarity rather than just a limitation would strengthen the narrative.
5. The rebuttal and supplementary material provide defocus and convergence angle series (Figs. S7-S9), demonstrating that the core lattice translation information remains robust. However, the manuscript itself does not offer practical recommendations for users. What defocus range is optimal for most applications? Is a larger convergence angle always preferable, or does the reduced depth of field compromise the visibility of the overlap region? Can image post-processing (e.g., focal series reconstruction) compensate for non-optimal parameters? These practical considerations would enhance the utility of the method for the broader community.

(Remarks on code availability)

Reviewer #3

(Remarks to the Author)
The authors used atomic resolution HAADF-STEM imaging and image simulations to reveal the nature of inclined stacking faults in fcc alloys, ordered inter metallic phases and GaP. The work primarily investigates the effects of electron channelling/dechanneling related to the displacement of the crystal within the thin foil specimen. With this, the authors are able to identify the translation vectors and types of various faults primarily in metallic alloys.

The experimental work appears to be of very high quality and the authors have carefully investigated the related image contrast with supporting image simulations. After a review stage, the manuscript seems to be significantly substantiated by imaging faults with different defocus values and semi-convergence angles to shed light on fault contrast in dependence of the imaging conditions. The authors have also extended their approach to another material system. The authors have also dedicated their time and resources to address the comments of the referee's and have with this largely increased the quality of the work. However, taking everything into consideration, I am coming to the same general conclusion that the work does not contain enough novelty to justify publication in Nature Communications.

Imaging of buried defects has been demonstrated previously by HAADF-STEM. In the end, the authors show that one can determine the displacement vector and with this type of the faults over a larger field of view, which seems to be important in the understanding of mechanical properties in superalloys. However, the same could be achieved by conventional diffraction contrast imaging. Possibly even with better statistics since the method used here still requires to capture atomic resolution images. Performing a detailed analysis of fringe contrast using conventional diffraction contrast imaging considering dynamical diffraction effects provides the same information as presented in the paper for these simple stacking fault configurations. What could be a novel aspect is to evaluate whether buried kinks or as outlined in the last section local segregation could be determined. The latter aspect would be a true advancement but as eluded by the authors is only included as an exploratory section. It is also not clear how quantitative this approach can be and may only be applicable to systems where heavy elements are distributed within the fault plane. It would certainly also be interesting if the presence of a nano cluster or precipitate could be revealed by conventional imaging simply by a local change in the structure factor? Simply claiming that the methodology will be expandable to other 2D defects is too far stretched, since for example grain boundaries involve way more complex lattice translation and rotation than stacking faults.

A true progress in imaging and analysis of inclined stacking faults could have been established by exploring novel imaging modalities like electron ptychography as outlined recently by Karapetyan et al. Nat Commun 2026 (https://doi.org/10.1038/s41467-026-69733-1). While electron ptyhography is limited to foil thicknesses of ~20 nm, it provides true 3D information with nm resolution and may even be used to explore strain relaxation and atomic redistribution.

In summary, the presented experimental work, associated image simulations and analysis are of very high quality but do not provide a major advancement, "only" a new angle and should be published in a more focused journal.

(Remarks on code availability)
I would suggest that peak finding and integration is done on the raw image. Image rotation requires pixel value interpolation and hence alters pixel intensities.

Version 1:

Reviewer comments:

Reviewer #2

(Remarks to the Author)
The authors have satisfactorily addressed all comments and concerns. In its current form, the manuscript is suitable for publication.

(Remarks on code availability)

Reviewer #3

(Remarks to the Author)
I thank the authors for their detailed reply.

I do not agree with the authors that they made a clear methodological advance. Comparing aberration corrected STEM images with multi-slice image simulations is a well established approach in the field used for many years. What the manuscript mainly does it applies it to stacking fault analysis in metallic alloys. Of course, the respective geometry of the fault inclination needs to be taken into account, but the authors apply a standard procedure to a very specific problem in Ni-based materials. Such work is certainly important to this research community, but in a broader context does not deliver new insights, besides the fault character. Which can also be determined by a much easier conventional TEM method not requiring an aberration-corrected STEM.

I doubt that the presented method is more robust than conventional TEM, because atomic resolution imaging of strongly bent samples will be equally difficult. The authors also never show a clear comparison where the conventional method would fail in comparison to the imaging method used here.

As I have mentioned earlier, being able to resolve local ordering or clustering of atoms at inclined faults would bring in a truly novel component to present a methodological advancement.

In general, the work would nicely fit into a microscopy oriented journal or for materials science where it addresses the corresponding readership.

(Remarks on code availability)

Open Access This Peer Review File is licensed under a Creative Commons Attribution 4.0 International License, which permits use, sharing, adaptation, distribution and reproduction in any medium or format, as long as you give appropriate credit to the original author(s) and the source, provide a link to the Creative Commons license, and indicate if changes were made.
In cases where reviewers are anonymous, credit should be given to 'Anonymous Referee' and the source.
The images or other third party material in this Peer Review File are included in the article’s Creative Commons license, unless indicated otherwise in a credit line to the material. If material is not included in the article’s Creative Commons

license and your intended use is not permitted by statutory regulation or exceeds the permitted use, you will need to obtain permission directly from the copyright holder.
To view a copy of this license, visit https://creativecommons.org/licenses/by/4.0/

Comments and Responses

Reviewer #1 (Remarks to the Author):

This revised version addresses previous comments with additional application examples and discussions, including on the effect of probe focus. Overall, the ability to observe stacking faults both inclined and edge on, as well as being able to indentify the nature of stacking faults in different type of crystal structures is valuable for materials characterization and represents a major use of aberration corrected transmission electron microscopy.

The manuscript is detailed, clearly written. However, the abstract and introduction part can be improved. The robustness of the method is not limited to the CoNi-based superalloy, for example, as the abstract would suggest. I would recommend publication after the authors address this point.

> Response (indented)
>
> Thank you for reviewing our revised manuscript again, and for your positive feedback and recommendation to publish.
>
> We have revised the introduction and rewritten the abstract to improve clarity and conciseness:
>
>> The nature of stacking faults (SF)—whether intrinsic or extrinsic—plays a pivotal role in defect-mediated processes in crystalline materials. Yet, transmission electron microscopy (TEM) techniques for their reliable analysis remain limited to either conventional fringe-contrast imaging of inclined faults or atomic-resolution imaging of edge-on configurations. Here, we overcome this long-standing geometric constraint by introducing a high-resolution scanning TEM method that enables full structural discrimination of inclined SFs, as demonstrated in fcc, $L1_2$, and sphalerite crystals. This approach complements edge-on analysis and provides access to SFs on all glide planes along commonly utilized zone axes. We demonstrate the method's robustness in a CoNi-based superalloy by discriminating fault types even in overlapping configurations and for foil thicknesses exceeding 100 nm, and extend its application to analyzing bounding partial dislocations in inclined geometries to reveal the fault formation mechanism. Probe propagation simulations reveal that fault-induced de-channeling is key to contrast formation and is strongly governed by the fault's depth within the sample. Leveraging this effect, we further establish a route to artificially generate ultrathin TEM lamellae—bounded by the SF itself—enhancing contrast for atomic-scale studies of long-range ordering and compositional fluctuations.

Reviewer #2 (Remarks to the Author):

The manuscript presents a HRSTEM-based methodology for analyzing inclined stacking faults in fcc, $L1_2$, and sphalerite crystal structures. By exploiting de-channeling contrast near the top surface of a TEM lamella, the authors demonstrate that the relative lattice translation across an inclined SF can be visualized, enabling discrimination between intrinsic and extrinsic faults. The method is applied to multiple material systems (CoNi-base superalloy, ODS alloy GRX-810, semiconductor GaP) and supported by multislice simulations. The extension to bounding partial dislocations via Burgers circuits and the observation of a "quasi-ultrathin" foil effect are also presented. This study addresses a relevant problem in defect analysis and contains interesting observations. However, in its current form, the manuscript does not yet provide sufficient evidence or discussion to warrant publication in Nature Communications.

Response (indented)

We thank the reviewer for recognizing that our study addresses a relevant problem in defect analysis, namely the discrimination between intrinsic and extrinsic faults in inclined geometries, and that the method is now demonstrated across multiple material systems and extended to the characterization of bounding partial dislocations. In response to the reviewer's comments (see below), we have revised the manuscript to more clearly present and substantiate the evidence and to clarify and streamline the discussion for the broad readership of *Nature Communications*.

Major comments:

1. While the proposed method is promising, several fundamental issues remain unaddressed: the non-unique determination of Burgers vectors, limited applicability, and overstated claims. Unless these concerns are resolved through a clear delineation of the method's capabilities and limitations, the work's impact will remain ambiguous.

Thank you for pointing out that the presentation could be made clearer with regard to these aspects. We note that these issues were already addressed in the previous version, albeit not always with sufficient clarity. Based on your detailed comments, we have revised the manuscript to more clearly delineate the scope, capabilities, and limitations of the method. Below, we respond to each point in detail and outline the corresponding revisions.

2. The authors demonstrate that for partial dislocations bounding a stacking fault, Burgers circuits can be drawn on HRSTEM images to obtain the projected Burgers vector (Fig. 9). However, even within this context, a limitation is not addressed: The Burgers vector obtained from a single zone axis is a two-dimensional projection and is non-unique in three-dimensional space. Different three-dimensional Burgers vectors can yield identical projections along a given zone

axis. This ambiguity is inherent to any single-projection analysis, yet the manuscript does not discuss how to resolve it. for example, by acquiring images along multiple zone axes or combining with complementary techniques. Given that the title emphasizes ’overcoming the edge-on limit‘ in defect analysis, this omission should be addressed.

We fully agree with the reviewer that a two-dimensional projection obtained from a single zone axis yields only a projected Burgers vector and is therefore not unique in three-dimensional space, and that this limitation is inherent to projection-based analyses. Accordingly, we have consistently distinguished between projected and full Burgers vectors throughout the manuscript. In this context, we also note that, in our earlier work (Acta Materialia 260 (2023), 119284), we determined the full Burgers vector of partial dislocations bounding stacking faults by combining two projections (⟨110⟩ and ⟨112⟩) accessed via a 30° sample tilt, both showing the stacking fault in edge-on orientation.

However, in the present work, the situation is different. The glide plane is inclined with respect to the viewing direction, and the dislocation line lies within this plane. Under the assumption of a glissile dislocation (i.e., with the Burgers vector contained in the glide plane), the projected Burgers vector uniquely defines the full Burgers vector. This is because the inclination sense of the glide plane can be directly inferred from the HRSTEM image through the overlapping lattice contrast at the top surface (as used in our method for stacking fault analysis), allowing reconstruction of both the magnitude and the sign of the Burgers vector.

We emphasize that this constitutes a key advantage of analyzing defects on inclined glide planes, as it enables determination of the full Burgers vector from a single projection, provided that crystallographic constraints (here, {111} glide planes in fcc-type materials) are taken into account and the dislocation can be reasonably assumed to be glissile. In contrast, when the glide plane is imaged edge-on, as is commonly the case, the Burgers vector component along the projection direction remains undetermined, even under the assumption of a glissile dislocation. This highlights that defect analysis in inclined geometries complements conventional high-resolution edge-on analysis and provides additional capabilities, particularly given that both geometries are typically present within a TEM lamella.

We note that this argument does not apply to the general case of non-glissile dislocations, where the Burgers vector is not confined to the glide plane. In the present case of the creep-deformed Co-based superalloy, the assumption of glissile dislocations is well justified and is further supported by the observed confinement of the dislocation line to the glide plane. However, whether the assumption of a glissile dislocation – and thus confinement of the Burgers vector to the inclined glide plane – is valid must be assessed on a case-by-case basis.

To address and clarify these important points raised by the reviewer, we have revised the manuscript as follows:

As can be seen in the magnified view in Fig. 9d, the Burgers circuit around the upper partial exhibits a closure failure equal to that partial's BV projected into the (001) image plane, $b_{1,proj}$, of $^{a}/_{6}$ [110] (cf. Supplementary Fig. 1). For the circuit around both partials, the closure failure is $^{a}/_{3}$ [110], representing the sum of both projected BVs $b_{1,proj} + b_{2,proj}$. Therefore, both projected BVs have identical values of $^{a}/_{6}$ [110]. **Given that the Burgers vector of a glissile dislocation lies in its glide plane, the true Burgers vector can be directly determined from this single projection:** In inclined fault geometry, there is an unambiguous correspondence between the true BV in the glide plane and its projection in the image plane (cf. Supplementary Fig. 1); this is an advantage over edge-on analysis, where different BVs **in a given glide plane** may have the same projection. Here, the projected values of $^{a}/_{6}$ [110] correspond to BVs b_1 and b_2 of $^{a}/_{6}$ $[11\bar{2}]$ in the (111) glide plane, i.e., both dislocations are identical Shockley partials. **While a conclusive determination of the BVs is possible for the glide process characterized here, it should be noted that, in the general case, an analysis based on a single projection may be ambiguous. This is especially relevant for dislocations moving by climb processes, where the BV may point out of the glide plane. A possible solution is to image the same fault configuration in two projections [47]**. The external compressive stress, which has acted in [001] direction perpendicular to the image plane, results in a force on the dislocations pointing upwards in Fig. 9a, i.e, these dislocations have lengthened the SF and are therefore confirmed to be its leading configuration.

Minor comments:

3. The method is demonstrated for cubic crystals and their derivatives. However, for HCP, stacking faults are predominantly confined to the basal plane. The authors mention in the rebuttal that the method is principle applicable, but this is not discussed. Does the method inherently rely on the presence of multiple inclined glide planes, and thus on high crystal symmetry? If so, this should be explicitly stated to avoid over-generalization.

We thank the reviewer for this important comment. The proposed method does not inherently rely on high crystal symmetry; however, it does require the presence of inclined glide planes in zone axes suitable for HRSTEM analysis (i.e., with sufficiently well-separated atomic columns). For hcp crystals, this condition is typically not fulfilled, as the most commonly accessible zone axes are those in which the basal plane is either oriented edge-on ($\langle 1\bar{1}00\rangle$, $\langle 11\bar{2}0\rangle$) or parallel ($\langle 0001\rangle$) to the foil surface. As a result, inclined geometries analogous to those exploited in cubic systems are generally not available or are of limited relevance.

More generally, the advantages of analyzing stacking faults on inclined planes become most pronounced in systems with multiple crystallographically equivalent glide planes. In contrast, in materials with a single dominant glide plane – such as the basal plane in hcp or layered

crystal structures – analysis in the conventional edge-on geometry remains the more appropriate approach. In such cases, the application of the present method does not provide additional benefit.

We have clarified this point in the revised manuscript to avoid overgeneralizing the added value of the method in such cases:

> Most importantly, when combined with the well-established HRSTEM analysis of edge-on SFs, the proposed method enables, for the first time, reliable analysis of SFs on all four {111} glide planes in a single sample using one microscopy technique. **Although the fcc and related structures are particularly suitable for this method owing to the presence of inclined glide planes in the projections commonly used for HRSTEM analysis,** the underlying principle is transferable to other crystal structures as well and is expected to facilitate defect characterization across a wide range of material systems, including high- and medium entropy alloys, steels, and thin-film systems.

4. The authors acknowledge that the method yields only the net translation vector and cannot resolve complex stacking sequences. This is presented as a limitation. However, the manuscript would benefit from a clearer statement that this positions the method as a complementary tool to edge-on HRSTEM, not a replacement. For studies requiring atomic-scale stacking sequences or chemical ordering within the fault plane, edge-on analysis remains indispensable. Framing this as a complementarity rather than just a limitation would strengthen the narrative.

We fully agree that the proposed method is best understood as complementary to conventional edge-on HRSTEM analysis and is intended to be used alongside it rather than as a replacement. As noted by the reviewer, edge-on imaging remains indispensable for resolving complex stacking sequences and for revealing segregation effects and local chemical ordering through STEM-EDXS compositional analysis, and thus represents the gold standard for the analysis of stacking fault chemistry. At the same time, not all glide planes are accessible in edge-on geometry (e.g., only two out of four in the common [110] zone axis of the fcc structure, and none in the [001] zone axis), necessitating characterization in inclined geometries. Under certain assumptions (as discussed above), the determination of full Burgers vectors of bounding partial dislocations represents an additional advantage. Taken together, edge-on and inclined geometries provide complementary information and can be combined within a single experiment, as they rely on the same imaging technique, namely HRSTEM.

The complementarity of both approaches is demonstrated in Fig. 5 of our manuscript through the simultaneous characterization of edge-on and inclined stacking faults within the same sample area in a [110] projection.

We appreciate the reviewer's suggestion to more clearly emphasize this complementarity and have revised the abstract, introduction, and summary of the manuscript accordingly:

Here, we overcome this long-standing geometric constraint by introducing a high-resolution scanning TEM method that enables full structural discrimination of inclined SFs, as demonstrated in fcc, $L1_2$, and sphalerite crystals. **This approach complements edge-on analysis and provides access to SFs on all glide planes along commonly utilized zone axes.** We demonstrate the method's robustness in a CoNi-based superalloy by discriminating fault types even in overlapping configurations and for foil thicknesses exceeding 100 nm, and extend its application to analyzing bounding partial dislocations in inclined geometries to reveal the fault formation mechanism.

(…)

To overcome these limitations, in this work, we present a new method enabling the analysis of SFs on inclined planes using HRSTEM (Fig. 1c), combining the advantages of this imaging technique with access to all four glide planes in both [001] and [110] zone axes (ZAs) **when used in combination with edge-on analysis**. To this end, projected structures of the different faults are investigated in the fcc and $L1_2$ structures in both [001] and [110] projection and compared to experimental HRSTEM micrographs acquired in a CoNi-base superalloy and corresponding multislice simulations.

(…)

Our novel approach to stacking fault (SF) analysis using high-resolution scanning transmission electron microscopy (HRSTEM) of inclined faults overcomes the long-standing limitations of the two classical SF analysis methods that have been employed for decades. **Directly applicable as a complementary tool to edge-on HRSTEM fault analysis,** the new method is highly flexible and robust in determining the intrinsic or extrinsic character of stacking faults, as demonstrated in both the fcc and $L1_2$ crystal structures of a compression-deformed superalloy, as well as an oxide-dispersion strengthened medium-entropy alloy and in a semiconductor specimen with sphalerite structure.

5. The rebuttal and supplementary material provide defocus and convergence angle series (Figs. S7-S9), demonstrating that the core lattice translation information remains robust. However, the manuscript itself does not offer practical recommendations for users. What defocus range is optimal for most applications? Is a larger convergence angle always preferable, or does the reduced depth of field compromise the visibility of the overlap region? Can image post-processing (e.g., focal series reconstruction) compensate for non-optimal parameters? These practical considerations would enhance the utility of the method for the broader community.

In our experience, a focus setting near the top surface of the foil yields optimal imaging conditions for the analysis, consistent with our simulations (Supplementary Fig. 9a). To achieve the best spatial resolution, the convergence angle should be chosen according to the optimum conditions of the electron optics. For the probe-corrected Thermo Fisher Spectra 200 (with CEOS Probe Corrector DCOR) operated at 200 kV, this corresponds to a convergence

angle of 30 mrad. At lower convergence angles, the resolution is limited by diffraction, whereas at higher angles, lens aberrations become increasingly dominant.

This optimum is also reflected in the image series shown in the Supplementary Fig. 9b, acquired with convergence angles of 15, 30, and 45 mrad. While stacking fault analysis is feasible in all cases, the image contrast is most favorable at 30 mrad.

Following the reviewer's suggestion, we have added practical recommendations to the main text:

> Given the inclined geometry of the SF in the foil, the location where the region of visible overlap appears in high-resolution micrographs varies slightly with defocus (see Supplementary Fig. 7 for an experimentally recorded defocus series). **The highest resolution, especially in the superposition region, is achieved with the focal plane near the top surface of the foil.** Furthermore, the width of this region depends on the effective depth of focus, and thus on the beam convergence angle (see Supplementary Fig. 8 for a direct comparison). **For the highest resolution, the optimal convergence angle for the specific microscope and correction optics should be used.** Both effects have been reproduced in multislice image simulations (Supplementary Fig. 9).

Reviewer #3 (Remarks to the Author):

The authors used atomic resolution HAADF-STEM imaging and image simulations to reveal the nature of inclined stacking faults in fcc alloys, ordered inter metallic phases and GaP. The work primarily investigates the effects of electron channelling/dechanneling related to the displacement of the crystal within the thin foil specimen. With this, the authors are able to identify the translation vectors and types of various faults primarily in metallic alloys.

Response (indented)

We would like to thank you for taking your time to review our revision, as well as the comments from the other reviewers, and for providing such detailed feedback. Below, we address each of the points raised.

The experimental work appears to be of very high quality and the authors have carefully investigated the related image contrast with supporting image simulations. After a review stage, the manuscript seems to be significantly substantiated by imaging faults with different defocus values and semi-convergence angles to shed light on fault contrast in dependence of the imaging conditions. The authors have also extended their approach to another material system. The authors have also dedicated their time and resources to address the comments of the referee's and have with this largely increased the quality of the work. However, taking everything into consideration, I am coming to the same general conclusion that the work does not contain enough novelty to justify publication in Nature Communications.

We thank the reviewer for the overall positive assessment of our manuscript and for acknowledging the extensive efforts undertaken in response to the reviewers' comments, which have substantially strengthened the quality and scope of the work.

We respectfully do not agree with the conclusion that the manuscript lacks sufficient novelty to justify publication in *Nature Communications*. As outlined in our previous responses and further substantiated in the revised version, the newly demonstrated capability to analyze inclined faults using HRSTEM represents a clear methodological advance beyond the current state. The approach is broadly applicable across materials systems and offers particularly strong advantages for FCC-type crystal structures where stacking faults play a central role, making it highly valuable for researchers working with various key classes of materials.

To support this, we emphasize: (i) the reliable and consistent analysis of both edge-on and inclined stacking faults within the same sample area (Fig. 5), using a single technique (HRSTEM) under identical imaging conditions (same zone axis, convergence angle, and focus setting on the top surface). In contrast, conventional approaches would require combining HRSTEM (for edge-on faults) with conventional TEM (for inclined faults), involving changes in imaging mode and extensive sample tilting to establish two-beam conditions; (ii) the robust analysis of heavily overlapping stacking faults in inclined geometries (Fig. 6c); and (iii) the characterization of complex or short stacking fault segments (e.g., in the ODS alloy, Fig. 7, as well as at the leading partials of a stacking fault, Fig. 9), both of which would be infeasible or at least extremely challenging using conventional TEM. In this context, we stress that the HRSTEM-based analysis is not restricted to very thin samples, as is typically used for high-resolution techniques, but can also be applied to specimens of thicknesses commonly used in conventional TEM studies. This is demonstrated for a superalloy sample (Fig. 6a,b, thickness > 100 nm) and a GaP semiconductor sample (Supplementary Fig. 11, thickness ~270 nm).

Taken together, these capabilities go clearly beyond existing methodologies and establish a broadly applicable framework for the analysis of crystalline defects. We therefore believe that the demonstrated methodological advance and its wide applicability meet the novelty expectations for publication in *Nature Communications*.

Imaging of buried defects has been demonstrated previously by HAADF-STEM.

The imaging of buried defects is a well-established capability of TEM and STEM techniques, including HAADF-STEM, and is not the focus of the present work. The key advance presented here is the ability to directly and reliably determine the fault type from a single HRSTEM micrograph in a straightforward manner and under a wide range of experimental conditions, as outlined above.

In the end, the authors show that one can determine the displacement vector and with this type of the faults over a larger field of view, which seems to be important in the understanding of mechanical properties in superalloys. However, the same could be achieved by conventional diffraction contrast imaging. Possibly even with better statistics since the method used here still requires to capture atomic resolution images. Performing a detailed analysis of fringe contrast using conventional diffraction contrast imaging considering dynamical diffraction effects provides the same information as presented in the paper for these simple stacking fault configurations.

As discussed in the Introduction, conventional fringe-contrast analysis poses its own challenges. In particular, under adverse conditions such as strongly bent crystals, short fault segments (which are often most relevant for deformation mechanisms), stacking faults overlapping in projection, or very thin foils, it becomes difficult or impossible to obtain fringe-contrast images that can be interpreted unambiguously.

In contrast, the analysis of HRSTEM micrographs remains reliable even under such conditions, as demonstrated in our manuscript. At the same time, we emphasize that this does not render conventional TEM-based approaches obsolete; rather, the capability to analyze inclined faults using HRSTEM complements existing methods and extends their applicability to regimes that are difficult to access by conventional diffraction-contrast imaging.

Furthermore, with recent advances in instrumentation, it is now easier than ever to record high-quality HRSTEM micrographs. The ability to use HRSTEM for the analysis of inclined faults therefore represents a clear methodological advance with practical relevance for a wide audience.

What could be a novel aspect is to evaluate whether buried kinks or as outlined in the last section local segregation could be determined. The latter aspect would be a true advancement but as eluded by the authors is only included as an exploratory section. It is also not clear how quantitative this approach can be and may only be applicable to systems where heavy elements are distributed within the fault plane. It would certainly also be interesting if the presence of a nano cluster or precipitate could be revealed by conventional imaging simply by a local change in the structure factor? Simply claiming that the methodology will be expandable to other 2D defects is too far stretched, since for example grain boundaries involve way more complex lattice translation and rotation than stacking faults.

We assume that the reviewer is referring to buried kinks in partial dislocations that bound stacking faults or separate intrinsic and extrinsic stacking-fault segments. Such kinks are extremely difficult to analyze in an edge-on orientation. It would therefore be very interesting to attempt their observation in an inclined geometry. However, we expect that even under inclined viewing conditions this remains highly challenging. Addressing this question would require a dedicated and comprehensive study, including simulations, and thus goes beyond both the scope and the thematic focus of the present work.

Regarding measurements of local segregation at planar faults, edge-on STEM analysis is generally the method of choice, particularly as it can be combined with EDX. However, in our exploratory study on the quasi-ultrathin foil effect, we demonstrate that superlattice ordering and lattice-site occupancies appear considerably more pronounced near inclined stacking faults than in bulk-type TEM samples, owing to this quasi-ultrathin foil effect. The presence of heavy alloying elements further facilitates the observation of these phenomena.

Exploring the quasi-ultrathin foil effect in greater detail is beyond the scope of the present study, but it represents a promising direction for future work. Regarding the extension to other two-dimensional defects, we did not intend to suggest that grain boundaries themselves could be analyzed in detail under inclined geometry. Rather, this passage was meant to refer to the emergence of the quasi-ultrathin foil effect. It is reasonable to expect that similar—if not stronger—dechanneling may occur at grain boundaries, in a manner analogous to stacking faults.

To further clarify this point, we have rephrased the corresponding sentence in our manuscript:

> These brief examples show that the contrast observed at inclined SFs provides opportunities for analysis even beyond characterization of the SF itself: the possibility to investigate atomic-column contrast from a region only a few nm thick without requiring an extremely thin foil may prove useful for future high-resolution studies, e.g., of long-range ordering, site occupancy, or clustering phenomena. Furthermore, **in the context of the quasi-ultrathin foil effect**, we suggest to also consider other two-dimensional defects such as grain or phase boundaries **as suitable sites for dechanneling**.

A true progress in imaging and analysis of inclined stacking faults could have been established by exploring novel imaging modalities like electron ptychography as outlined recently by Karapetyan et al. Nat Commun 2026 (https://doi.org/10.1038/s41467-026-69733-1). While electron ptyhography is limited to foil thicknesses of ~20 nm, it provides true 3D information with nm resolution and may even be used to explore strain relaxation and atomic redistribution.

Ptychography is indeed a powerful and rapidly advancing technique, as highlighted by the reviewer, offering access to three-dimensional information at high spatial resolution. At the same time, it requires highly specialized data acquisition and reconstruction procedures, as well as very thin specimens (on the order of ~20 nm), which can limit its applicability in many experimental contexts.

In contrast, the approach presented in our work is conceived with a different objective. It enables the analysis of inclined stacking faults through direct HRSTEM imaging, requiring only a single micrograph and no elaborate reconstruction. Rather than aiming at comprehensive 3D characterization, our method focuses on providing direct, efficient access to relevant structural information under broadly accessible experimental conditions.

We therefore view the two approaches as complementary, with clearly distinct scopes and strengths: while ptychography excels in delivering detailed three-dimensional insights under highly controlled conditions, our method offers a conceptually straightforward and experimentally versatile route to the analysis of inclined defects.

In summary, the presented experimental work, associated image simulations and analysis are of very high quality but do not provide a major advancement, “only” a new angle and should be published in a more focused journal.

We appreciate the reviewer’s positive assessment of the quality of our work. Following up on our responses above, it is precisely this “new angle” that represents a key advancement in fault analysis. In complement to established edge-on approaches, it now enables the straightforward and simultaneous HRSTEM analysis of stacking faults on all four glide planes in fcc structures and beyond. We are confident that this capability constitutes a significant advance that merits publication in *Nature Communications*.

Reviewer #3 (Remarks on code availability):

I would suggest that peak finding and integration is done on the raw image. Image rotation requires pixel value interpolation and hence alters pixel intensities.

Thank you for this suggestion. We will look into a possible improvement of the code in this regard.

Response to comments by Reviewer #2:

Response (indented)

The authors have satisfactorily addressed all comments and concerns. In its current form, the manuscript is suitable for publication.

We sincerely thank Reviewer #2 for the careful evaluation of our manuscript throughout the review process. We greatly appreciate the reviewer's final assessment that all comments and concerns have been satisfactorily addressed and that the manuscript, in its current form, is suitable for publication in *Nature Communications*. We are pleased that the extensive revisions, additional experiments, image simulations, and robustness studies performed during the review process have successfully resolved all issues raised by the reviewer.

Response to comments by Reviewer #3:

The comments raised by Reviewer #3 largely concern issues that were extensively discussed during the previous review rounds and had already been addressed through substantial revisions of the manuscript. Consequently, where appropriate, we refer to the corresponding additions incorporated into the previous revision rather than repeating our previous responses in full.

I thank the authors for their detailed reply.

I do not agree with the authors that they made a clear methodological advance. Comparing aberration corrected STEM images with multi-slice image simulations is a well established approach in the field used for many years. What the manuscript mainly does it applies it to stacking fault analysis in metallic alloys. Of course, the respective geometry of the fault inclination needs to be taken into account, but the authors apply a standard procedure to a very specific problem in Ni-based materials. Such work is certainly important to this research community, but in a broader context does not deliver new insights, besides the fault character.

This concern was extensively addressed during the previous revision rounds through substantial additions to the manuscript, including additional application examples, robustness studies, expanded analyses of imaging conditions, and demonstrations in different material systems and crystal structures.

Regarding the methodological contribution, we respectfully disagree with the reviewer's assessment. The central advance of the present work is not the comparison of HRSTEM images with multislice simulations, which is indeed a well-established approach. Rather, the methodological advance lies in the development and validation of a robust method for analyzing inclined stacking faults by high-resolution STEM, thereby overcoming the long-standing edge-on limitation in atomic-scale defect characterization. The multislice simulations serve to validate and rationalize the observed contrast mechanisms but are not the primary methodological contribution.

In the previous review round, Reviewer #3 explicitly acknowledged the application of the methodology "to reveal the nature of inclined stacking faults in fcc alloys, ordered intermetallic phases and GaP." It is therefore difficult to reconcile this assessment with

the present statement that "the authors apply a standard procedure to a very specific problem in Ni-based materials."

In fact, the revised manuscript demonstrates that the methodology is not restricted to Ni-based alloys or metallic materials. It includes applications to different fault configurations, crystal structures, and material classes, including the semiconductor GaP. Furthermore, the method provides information beyond the fault character alone; Fig. 9 demonstrates characterization of the partial dislocations bounding the fault, thereby providing insight into the formation mechanism of the defect. These examples were already included in the version evaluated in the present review.

Which can also be determined by a much easier conventional TEM method not requiring an aberration-corrected STEM.

I doubt that the presented method is more robust than conventional TEM, because atomic resolution imaging of strongly bent samples will be equally difficult.

This point essentially reiterates a concern raised in the reviewer's previous report, where it was argued that "the same could be achieved by conventional diffraction contrast imaging." We addressed this point in detail in our previous response by explaining why the HRSTEM-based methodology provides distinct advantages over conventional diffraction-contrast TEM in a number of practically important situations. Rather than repeating our previous discussion, we focus here on the reviewer's specific statement that "I doubt that the presented method is more robust than conventional TEM, because atomic resolution imaging of strongly bent samples will be equally difficult."

The robustness of the HRSTEM-based method arises from the highly localized nature of the information used for defect analysis. Reliable interpretation requires only a very small region of the specimen (typically less than 10 nm adjacent to the intersection of the stacking fault with the specimen surface; see the examples throughout the manuscript) to be oriented along a zone axis, whereas conventional diffraction-contrast TEM requires suitable diffraction conditions over a substantially larger specimen area. Local orientation variations caused by specimen bending therefore affect conventional TEM much more strongly and can render defect analysis difficult or ambiguous.

In order to clarify this point, we added the following paragraph to Section 2.3 ("Advantages and Practical Considerations") of the revised manuscript:

> More generally, the robustness of the proposed HRSTEM method for SF analysis benefits from its highly localized evaluation of the image contrast. This advantage is not limited to the analysis of overlapping SFs but also extends to specimens exhibiting local foil bending, a common phenomenon in TEM samples and therefore of considerable practical relevance. Since only a small region (typically less than 10 nm) adjacent to the intersection of the SF with the specimen surface needs to be aligned to the desired zone axis, moderate local orientation variations have little influence on the analysis. In contrast, conventional CTEM approaches based on SF fringe contrast require suitable diffraction conditions to be maintained over much larger regions, typically on the order of several tens to hundreds of nanometers. Local foil bending can therefore lead to significant variations in the diffraction conditions across the analyzed area, resulting in corresponding changes in the fringe contrast and making the interpretation considerably less reliable.

Importantly, the advantages of the HRSTEM method are not limited to bent specimens. As demonstrated in the manuscript, the proposed approach also enables reliable

analysis of faults overlapping in projection (Fig. 6), small fault segments associated with stacking-fault nodes (Fig. 7), and fault segments only a few nanometers in length that are directly involved in a defect formation mechanism (Fig. 9). In all of these situations, conventional diffraction-contrast TEM faces significant limitations in providing the same level of unambiguous structural information. A direct comparison between conventional TEM and the proposed HRSTEM-based approach for faults overlapping in projection is provided in Supplementary Fig. 6.

Finally, recent advances in aberration-corrected STEM instrumentation have made high-quality atomic-resolution imaging routinely accessible for materials characterization. The ability to extend HRSTEM to the reliable analysis of inclined stacking faults therefore represents not only a methodological advance but also a practically relevant extension of the capabilities of modern electron microscopy. Moreover, the revised manuscript demonstrates that the methodology remains robust over a wide range of convergence semi-angles, including values representative of conventional, non-aberration-corrected STEM operation. This demonstrates that the methodology is not intrinsically dependent on aberration correction.

The authors also never show a clear comparison where the conventional method would fail in comparison to the imaging method used here.

Contrary to the reviewer's statement, the revised manuscript does contain a direct comparison between conventional diffraction-contrast TEM and the proposed HRSTEM-based approach. Supplementary Fig. 6 presents the case of stacking faults overlapping in projection, where the HRSTEM-based method yields an unambiguous result, whereas conventional diffraction-contrast analysis does not. This comparison was specifically included to address the relative capabilities and limitations of the two approaches.

As I have mentioned earlier, being able to resolve local ordering or clustering of atoms at inclined faults would bring in a truly novel component to present a methodological advancement.

We respectfully disagree that the methodological advance presented in this work depends on resolving local ordering or clustering at inclined faults.

The central contribution of the manuscript is the development and validation of a robust method for the reliable analysis of inclined stacking faults by high-resolution STEM, thereby overcoming the long-standing edge-on limitation in atomic-scale defect characterization.

The quasi-ultrathin foil effect arises as an additional consequence of the dechanneling mechanism exploited for stacking-fault analysis. As demonstrated in the manuscript, this effect gives rise to a quasi-ultrathin imaging condition near the intersection of the stacking fault with the specimen surface, enhancing the visibility of local ordering and compositional variations. These effects are documented and statistically substantiated in the manuscript, including a first example of a nanocluster revealed by this mechanism (Supplementary Fig. 13).

A comprehensive investigation of ordering and clustering phenomena enabled by this effect would constitute a substantial research topic in its own right and lies beyond the scope of the present work. Importantly, the methodological advance established here

does not depend on such future studies, but on the reliable characterization of inclined stacking faults themselves enabled by the proposed methodology.

In general, the work would nicely fit into a microscopy oriented journal or for materials science where it addresses the corresponding readership.

We respectfully submit that the manuscript has broader significance. It establishes a generally applicable methodology for overcoming the edge-on limitation in atomic-scale defect analysis, demonstrates robustness across different imaging conditions, and applies the approach to multiple classes of materials.

This view is consistent with the final assessments of the two original reviewers, who evaluated the manuscript throughout the review process and whose comments formed the basis for substantial revisions. They ultimately concluded that their concerns had been satisfactorily addressed. Their recommendation for publication in *Nature Communications* demonstrates that they considered the work to be both sufficiently significant and of sufficiently broad interest for the journal.
